# Data on unveiling the occurrence of transient, multi-contaminated mafic magmas inside a rhyolitic reservoir feeding an explosive eruption (Nisyros, Greece)

**DOI:** 10.1016/j.dib.2022.108077

**Published:** 2022-03-23

**Authors:** F. Mastroianni, E. Braschi, M. Casalini, S. Agostini, S. Di Salvo, G. Vougioukalakis, L. Francalanci

**Affiliations:** a Dipartimento di Scienze della Terra, Università di Pisa, Via S. Maria, 53, Pisa 56126, Italy; b Dipartimento di Scienze della Terra, Università degli studi di Firenze, Via Giorgio La Pira, 4, Firenze 50121, Italy; c Istituto di Geoscienze e Georisorse, Consiglio Nazionale delle Ricerche, sede secondaria di Firenze, Via Giorgio La Pira, 4 50121, Italy; d Istituto di Geoscienze e Georisorse, Consiglio Nazionale delle Ricerche, sede di Pisa, Via G. Moruzzi, 1 56124, Italy; e Hellenic Survey of Geology and Mineral Exploration, 3rd Entrance Olympic village 13637, Aharne, Athens, Greece

**Keywords:** Nisyros, Upper Pumice eruption, Sr-Nd isotopes, Mineral chemistry, Undercooling textures

## Abstract

This data article includes the description and the geochemical and mineralogical dataset of 67 pyroclastic rock samples from the Upper Pumice (UP) explosive activity of Nisyros volcano (eastern South Aegean Active Volcanic Arc). A detailed field and petrographic description of the studied outcrops and samples are reported, including representative photomicrographs and SEM images, whole-rock major and trace elements compositions of 31 representative samples and Sr-Nd isotope ratios on 22 selected samples. Analytical methods and conditions used for data acquisition are also reported.

The UP eruption produced a stratigraphic sequence constituted by a basal fallout deposit, gradually substituted by pyroclastic density current (PDC) deposits; these are overlaid by a lag-breccia unit, and the sequence is closed by a grey ash flow level. The juvenile is mainly constituted by white-yellow, moderately crystalline pumice with rhyolitic composition and homogenous Sr-Nd isotope values. Variable amounts of dense, grey, crystalline juvenile lapilli clasts (CRC, Crystal-Rich Clast), with rounded shape and less evolved composition (andesite to dacite) are also present in the deposit. These mafic CRCs are peculiar due to their large variability in textures (from distinctive diktytaxitic to strongly fragmented structure without a defined fabric) and in the geochemical and isotopic composition.

The data acquired were fundamental to reconstruct the complex and peculiar history of ascent, storage and differentiation/assimilation processes of these mafic melts before their intrusion into the shallow, rhyolitic magma chamber, with important implication on the possible eruption trigger during the more recent explosive phase of activity at Nisyros volcano. Moreover, the geochemical and isotopic analyses provide new original data to the general knowledge of the Aegean volcanics.

All the data reported in this paper are related to the research article Braschi et al. (2022)

## Specifications Table

**Table:** 

Subject area	Earth and Planetary Sciences
Specific subject area	Geochemistry and Petrology
Type of data	Text files, pictures, tables and graphs.
How data was acquired	Field work: detailed sampling and deposit description during two field campaigns. Petrographic analyses through polarized light microscopy. Image acquisition through scanning electron microscope (SEM). Laboratory measurements: Sr isotope composition through Thermal Ionization Mass Spectrometry (TIMS) and Nd isotope composition through Multicollector Inductively Coupled Plasma Mass Spectrometry (MC-ICP-MS). Major and minor composition of mineral phases through Electron Microprobe Analysis (EPMA).
Data format	Raw and analysed
Description of data collection	All data are originals and were collected by the authors using accepted procedures and robust analytical condition. Details of data collection are reported in the “Experimental Design, Materials, and Methods” section.
Data source location	Institution: Istituto di Geoscienze e Georisorse, Sezione di Firenze; Dipartimento di Scienze della Terra, Università degli Studi di Firenze. City/Town/Region: Firenze Country: Italy Latitude and longitude (and GPS coordinates, if possible) for collected samples/data: 36.58905° N, 27.16918° E Nisyros Island, Dodecanese, Greece
Data accessibility	Repository name: EarthChem Library Data identification number (permanent identifier, i.e. DOI number): DOI: 10.26022/IEDA/112,230. Direct link to the dataset: https://ecl.earthchem.org/view.php?id=2230.
Related research article	E. Braschi, F. Mastroianni, S. Di Salvo, M. Casalini, S. Agostini, G. E. Vougioukalakis, L. Francalanci, Unveiling the occurrence of transient, multi-contaminated mafic magmas inside a rhyolitic reservoir feeding an explosive eruption (Nisyros, Greece), Lithos 410–411 (2022) 106,574. https://doi.org/10.1016/j.lithos.2021.106574

## Value of the Data

• These data are crucial for the reconstruction of the plumbing system dynamic of Nisyros Volcano before the Upper Pumice eruption.
• The data, including mineral chemistry and Sr-Nd isotope ratios, expand and integrate the existent database of volcanic products of the South Aegean Active Volcanic Arc.
• Crystal-rich clasts (CRC) show the lowest 143Nd/144Nd values recorded for the Nisyros-Kos-Yali volcanic field.
• The data will contribute to a better understanding of the involvement of different crustal components and ascent pathways of mafic magmas below active volcanoes in subduction zones.

## 1 Data and Images

Data, images and figures here reported were interpreted and discussed in Braschi et al. [1] to unravel the origin and evolution of the mafic components erupted by the UP activity, and their interaction with the main rhyolitic host magma. The full dataset of major, trace elements and Sr-Nd isotopes on whole rocks, together with glass composition and mineral chemistry is available in the EarthChem Library repository at https://doi.org/10.26022/IEDA/112230.

### 1.1 Field observation

**Fig. 1:**
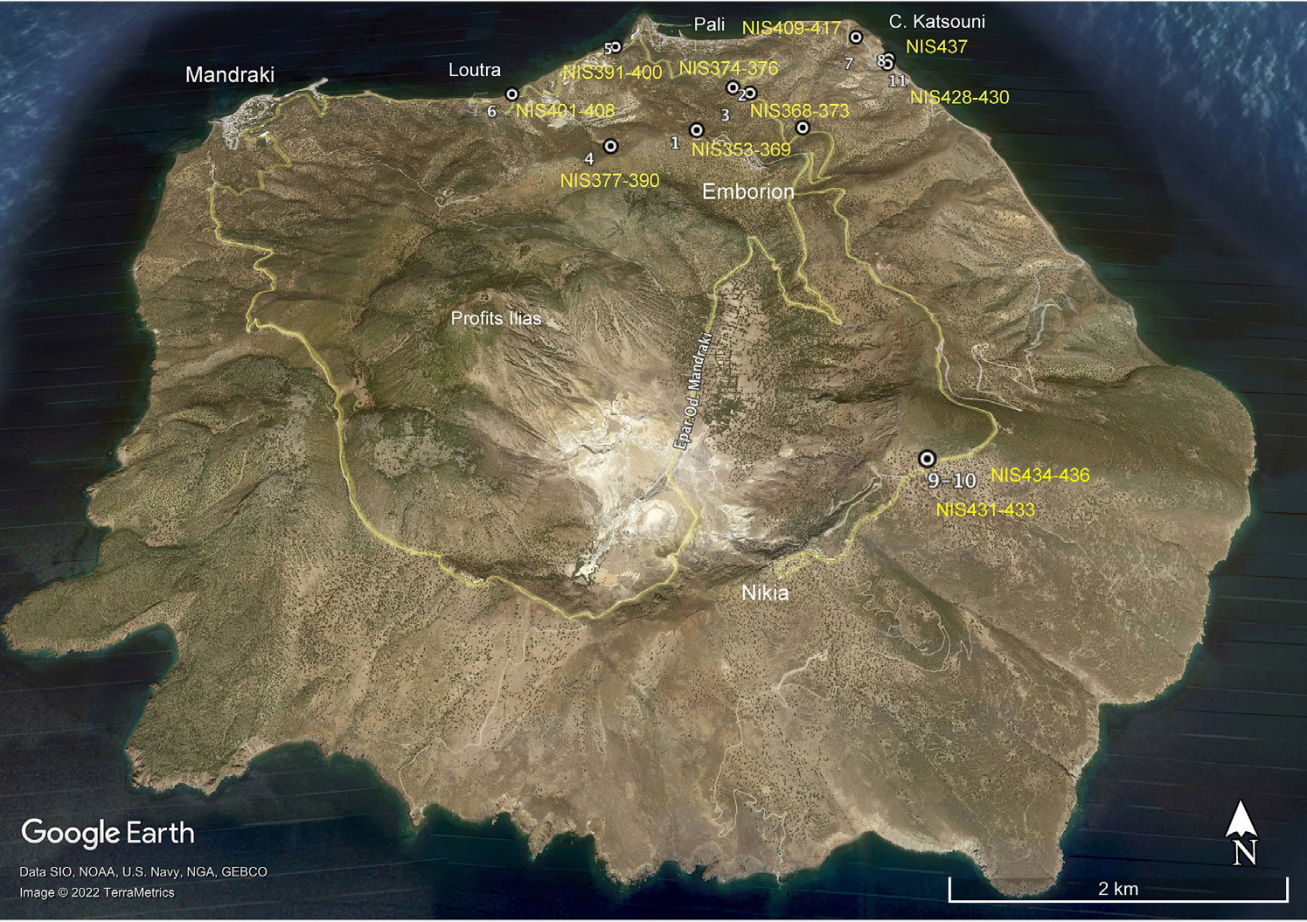
Location of the collected samples.

Table 1 is a list of the samples collected from the Upper Pumice (UP) deposit. The table reports detailed information of the sampling locations for the different outcrops (see also Fig. 1), including the type of depositional unit. A schematic petrographic description of each sample is also reported including their structure, paragenesis and texture features. Some samples have been subdivided into different portions according to their characteristics and labelled with different letters.

**Table 1:** Location and petrographic description of the studied samples from the Upper Pumice deposit (Nisyros, Greece).

Sample	Sampling location	Coordinates		Elevation m s.l.m.	Outcrop	Depositional Unit	Lithology	Sample size	Sample Texture	CRC Texture Type	Paragenesis	plg/femic	Cristallinity (vol. %)	Vacuolarity (vol. %)	Glass (vol. %)	Crystal Aggregates	(Micro-) Enclaves	Reaction rim	ph+mph in CRC (vol. %)
**NIS312**	Cape Katzouni	36°36′55.38″N	27°11′25.52″E	18	8	Lag-breccia	Crystal rich clast	ca. 20 cm		Type-C	Plg, Cpx, Ox, Ol, Opx, Amph	65/35	45	45	10	✓		✓	1
**NIS313**	Cape Katzouni	36°36′55.38″N	27°11′25.52″E	18	8	Lag-breccia	Crystal rich clast			Type-C	Plg, Cpx, Ox, Opx, Amph	35/35	45	40	15	✓			5
**NIS314**	Cape Katzouni	36°36′55.38″N	27°11′25.52″E	18	8	Lag-breccia	Crystal rich clast			Type-B	Plg, Cpx, Ox, Ol, Amph	55/45	37	53	10	✓		✓	10
**NIS315**	Pali - main road	36°37′0.10″N	27° 9′57.52″E	38	5	Fallout	Pumice		porphyritic		Plg, Opx, Cpx, Amph, Ox	90/10	5	50	45				
**NIS316a**	Pali - main road	36°37′0.10″N	27° 9′57.52″E	38	5	Fallout	Crystal rich clast			Type-A	Plg, Cpx, Ox, Ol, Opx, Amph	60/40	40	50	10		✓		20
**NIS316b**	Pali - main road	36°37′0.10″N	27° 9′57.52″E	38	5	Fallout	Crystal rich clast			Type-B	Plg, Cpx, Ox, Opx, Amph	60/40	45	45	10		✓		15
**NIS316c**	Pali - main road	36°37′0.10″N	27° 9′57.52″E	38	5	Fallout	Crystal rich clast			Type-B	Plg, Cpx, Ox, Opx, Amph	90/10	40	45	15		✓		7
***NIS316d***	Pali - main road	36°37′0.10″N	27° 9′57.52″E	38	5	Fallout	Crystal rich clast			Type-B	Plg, Cpx, Ox, Opx, Amph	85/15	38	52	10		✓	✓	1
***NIS316e***	Pali - main road	36°37′0.10″N	27° 9′57.52″E	38	5	Fallout	Crystal rich clast			Type-B	Plg, Cpx, Ox, Opx, Amph	95/5	15	65	20				1
***NIS316f***	Pali - main road	36°37′0.10″N	27° 9′57.52″E	38	5	Fallout	Crystal rich clast			Type-A	Plg, Cpx, Ox, Opx, Amph	80/20	30	60	10				
***NIS316g***	Pali - main road	36°37′0.10″N	27° 9′57.52″E	38	5	Fallout	Crystal rich clast			Type-A	Plg, Cpx, Ox, Opx, Amph	70/30	30	50	20				1
***NIS316h***	Pali - main road	36°37′0.10″N	27° 9′57.52″E	38	5	Fallout	Crystal rich clast			Type-B	Plg, Cpx, Ox, Opx, Amph	90/10	30	55	15		✓		5
**NIS 317**	Emborion cemetery	36°36′20.06″N	27°10′21.90″E	362	1	Fallout	Crystal rich clast			Type-A	Plg, Cpx, Ox, Opx, Amph	90/10	30	50	20				
**NIS 318**	Emborion cemetery	36°36′20.06″N	27°10′21.90″E	362	1	Fallout	Crystal rich clast			Type-B	Plg, Cpx, Ox, Opx, Amph	70/30	25	55	20				1
**NIS 353**	Emborion cemetery	36°36′20.06″N	27°10′21.90″E	362	1	Fallout	Pumice	5–10 cm	porphyritic		Plg, Opx, Ox, Amph	75/25	15	30	55	✓	✓		
**NIS 354**	Emborion cemetery	36°36′20.06″N	27°10′21.90″E	362	1	Fallout	Pumice												
**NIS 355**	Emborion cemetery	36°36′20.06″N	27°10′21.90″E	362	1	Fallout	Crystal rich clast	ca. 20 cm	vesicular, aphyric	Type-A	Plg, Cpx, Ox, Ol, Opx, Amph	60/40	45	40	15	✓		✓	10
**NIS 356**	Emborion cemetery	36°36′20.06″N	27°10′21.90″E	362	1	Fallout	Crystal rich clast	ca. 15 cm	high vesicular, aphyric/microcrystalline	Type-A	Plg, Opx, Cpx, Amph, Ox	65/26	35	50	15	✓			30
**NIS 357**	Emborion cemetery	36°36′20.06″N	27°10′21.90″E	362	1	Fallout	Crystal rich clast	ca. 25 cm	low vesicular, aphyric	Type-B	Plg, Opx, Cpx, Amph, Ox	75/25	55	25	20	✓		✓	10
**NIS 358**	Emborion cemetery	36°36′20.06″N	27°10′21.90″E	362	1	Fallout	Crystal rich clast	ca. 15 cm	vesicular, aphyric/microcrystalline	Type-A	Plg, Opx, Cpx, Amph, Ox	70/30	40	45	15			✓	1
NIS 358*	Emborion cemetery	36°36′20.06″N	27°10′21.90″E	362	1	Fallout	Pumice	ca. 15 cm	porphyritic		Plg, Opx, Cpx, Amph, Ox	80/20	10	50	40		✓		
**NIS 359**	Emborion cemetery	36°36′20.06″N	27°10′21.90″E	362	1	Fallout	Crystal rich clast	30–40 cm	low vesicular, aphyric	Type-A	Plg, Opx, Cpx, Amph, Ox	70/30	38	45	17	✓		✓	5
**NIS 360**	Emborion cemetery	36°36′20.06″N	27°10′21.90″E	362	1	Fallout	Crystal rich clast	5–8 cm	vesicular, microcrystalline	Type-B	Plg, Opx, Cpx, Amph, Ox	80/20	45	37	18	✓			5
**NIS 361**	Emborion cemetery	36°36′20.06″N	27°10′21.90″E	362	1	Fallout	Crystal rich clast	ca. 30 cm	low vesicular, aphyric	Type-A	Plg, Cpx, Ox, Ol, Opx, Amph	50/50	40	45	15				5
**NIS 362**	Emborion cemetery	36°36′20.06″N	27°10′21.90″E	362	1	Fallout	Crystal rich clast			Type-B	Plg, Cpx, Ox, Ol, Opx, Amph	60/40	40	50	10		✓	✓	1
**NIS 363**	Emborion cemetery	36°36′20.06″N	27°10′21.90″E	362	1	Fallout	Crystal rich clast			Type-B	Plg, Cpx, Ox, Ol, Opx, Amph	60/40	25	70	5		✓	✓	5
**NIS 364**	Emborion cemetery	36°36′20.06″N	27°10′21.90″E	362	1	Fallout	Crystal rich clast			Type-B	Plg, Cpx, Ox, Ol, Opx, Amph	70/30	30	50	20		✓	✓	5
**NIS 365**	Emborion cemetery	36°36′20.06″N	27°10′21.90″E	362	1	Fallout	Crystal rich clast			Type-B	Plg, Cpx, Ox, Ol, Opx, Amph	80/20	40	40	20		✓		7
**NIS 366**	Emborion cemetery	36°36′20.06″N	27°10′21.90″E	362	1	Fallout	Crystal rich clast			-	-	-	-	-	-	-	-	-	-
**NIS 367**	Emborion cemetery	36°36′20.06″N	27°10′21.90″E	362	1	Fallout	Crystal rich clast			-	-	-	-	-	-	-	-	-	-
**NIS 368**	Emborion cemetery	36°36′20.06″N	27°10′21.90″E	362	1	Fallout	Crystal rich clast			Type-B	Plg, Cpx, Ox, Ol, Opx, Amph	75/25	40	45	15				10
**NIS 370**	Main road	36°36′38.78″N	27°10′39.86″E	151	2	Fallout	Crystal rich clast	30–40 cm	vesicular, aphyric/microcrystalline	Type-A	Plg, Opx, Amph, Ox	70/30	30	60	10				10
**NIS 371**	Main road	36°36′38.78″N	27°10′39.86″E	151	2	Fallout	Crystal rich clast	ca. 30 cm	vesicular, aphyric	Type-A	Plg, Opx, Cpx, Amph, Ox	70/30	32	56	12	✓			10
**NIS 372b**	Main road	36°36′38.78″N	27°10′39.86″E	151	2	Fallout	Crystal rich clast		low vesicular, aphyric/microcrystalline	Type-C	Plg, Cpx, Ox, Ol, Opx, Amph	60/40	45	45	10		✓	✓	3
**NIS 374**	Lateral valley	36°36′40.55″N	27°10′34.57″E	159	3	Fallout	Pumice		porphyritic		Plg, Opx, Cpx, Amph, Ox	93/7	7	40	53	✓	✓		
**NIS 375**	Lateral valley	36°36′40.55″N	27°10′34.57″E	159	3	Fallout	Crystal rich clast		high vesicular, aphyric/microcrystalline	Type-A	Plg, Opx, Cpx, Amph, Ox	70/30	30	60	10				5
**NIS 377**	Caldera rim	36°36′16.64″N	27° 9′56.06″E	323	4	Fallout	Pumice	6–30 cm	porphyritic		Plg, Opx, Cpx, Amph, Ox	80/20	10	35	55	✓	✓		
**NIS 378**	Caldera rim	36°36′16.64″N	27° 9′56.06″E	323	4	Fallout	Crystal rich clast	ca. 40 cm	vesicular, aphyric/microcrystalline	Type-A	Plg, Cpx, Ox, Ol, Amph	60/40	50	30	20	✓			10
NIS 378*	Caldera rim	36°36′16.64″N	27° 9′56.06″E	323	4	Fallout	Pumice	ca. 40 cm	porphyritic		Plg, Opx, Amph, Ox	90/10	5	45	50	✓	✓		
**NIS 379b**	Caldera rim	36°36′16.64″N	27° 9′56.06″E	323	4	Fallout	Crystal rich clast	ca. 10 cm	low vesicular, microcrystalline	Type-C									
**NIS 380**	Caldera rim	36°36′16.64″N	27° 9′56.06″E	323	4	Fallout	Crystal rich clast	ca. 30 cm	low vesicular, aphyric/microcrystalline	Type-A	Plg, Opx, Cpx, Amph, Ox	65/35	25	60	15		✓		
**NIS 381**	Caldera rim	36°36′16.64″N	27° 9′56.06″E	323	4	Fallout	Crystal rich clast	ca. 40 cm	low vesicular, aphyric	Type-A	Plg, Cpx, Ox, Ol, Opx, Amph	75/25	38	42	20				15
NIS 381*	Caldera rim	36°36′16.64″N	27° 9′56.06″E	323	4	Fallout	Pumice	ca. 40 cm	porphyritic		Plg, Cpx, Ox, Ol, Opx, Amph	85/15	10	40	50	✓	✓		
**NIS 383**	Caldera rim	36°36′16.64″N	27° 9′56.06″E	323	4	Fallout	Pumice		porphyritic, banded	Type-A	Plg, Cpx, Ox, Ol, Opx, Amph	65/35	40	45	15				10
**NIS 385**	Caldera rim	36°36′16.64″N	27° 9′56.06″E	323	4	Fallout	Crystal rich clast		low vesicular, aphyric/microcrystalline	Type-A	Plg, Opx, Cpx, Amph, Ox	70/30	40	45	15				2
**NIS 390**	Caldera rim	36°36′16.64″N	27° 9′56.06″E	323	4	Fallout	Pumice		banded										
**NIS 401**	Loutra - Gas station	36°36′43.67″N	27° 9′24.27″E	35	6	Fallout	Pumice		porphyritic		Plg, Cpx, Ox, Ol, Opx, Amph	96/4	10	40	50	✓	✓		
**NIS 402**	Loutra - Gas station	36°36′43.67″N	27° 9′24.27″E	35	6	Fallout	Pumice												
**NIS 409**	Road to Cape Katzouni	36°37′2.19″N	27°11′17.11″E	34	7a	Lag-breccia	Pumice												
**NIS 413**	Road to Cape Katzouni	36°37′2.19″N	27°11′17.11″E	34	7a	Lag-breccia	Crystal rich clast	2–3 cm		Type-C	Plg, Cpx, Ox, Ol, Opx, Amph	80/20	45	45	10				2
**NIS 416**	Road to Cape Katzouni	36°37′2.19″N	27°11′17.11″E	34	7a	Lag-breccia	Crystal rich clast	>20 cm	vesicular, microcrystalline	Type-C	Plg, Cpx, Ox, Opx, Amph	70/30	35	55	10	✓			10
**NIS 418**	Cape Katzouni	36°36′55.38″N	27°11′25.52″E	18	8	Lag-breccia	Pumice	ca. 50 cm	porphyritic		Plg, Cpx, Ox, Opx, Amph	93/7	5	52	43			✓	
**NIS 419**	Cape Katzouni	36°36′55.38″N	27°11′25.52″E	18	8	Lag-breccia	Pumice	ca. 10 cm	porphyritic		Plg, Cpx, Ox, Ol, Opx, Amph	93/7	10	50	40	✓	✓		
**NIS 420b**	Cape Katzouni	36°36′55.38″N	27°11′25.52″E	18	8	Lag-breccia	Crystal rich clast	4–10 cm	vesicular, aphyric/microcrystalline	Type-A	Plg, Cpx, Ox, Opx, Amph	85/15	45	40	15				5
**NIS 421**	Cape Katzouni	36°36′55.38″N	27°11′25.52″E	18	8	Lag-breccia	Crystal rich clast	>40 cm	low vesicular, microcrystalline	Type-C	Plg, Cpx, Ox, Opx, Amph	70/30	50	40	10	✓		✓	15
**NIS 422**	Cape Katzouni	36°36′55.38″N	27°11′25.52″E	18	8	Lag-breccia	Crystal rich clast	>40 cm	low vesicular, microcrystalline	Type-C	Plg, Cpx, Ox, Opx, Amph	65/35	50	40	10	✓		✓	
**NIS 423**	Cape Katzouni	36°36′55.38″N	27°11′25.52″E	18	8	Lag-breccia	Crystal rich clast	ca. 15 cm	vesicular, microcrystalline	Type-B	Plg, Cpx, Ox, Ol, Opx, Amph	80/20	35	55	10	✓			15
**NIS 424**	Cape Katzouni	36°36′55.38″N	27°11′25.52″E	18	8	Lag-breccia	Crystal rich clast	ca. 40 cm	vesicular, microcrystalline	Type-B	Plg, Cpx, Ox, Ol, Opx, Amph	65/35	40	45	15	✓		✓	5
**NIS 425**	Cape Katzouni	36°36′55.38″N	27°11′25.52″E	18	8	Lag-breccia	Crystal rich clast		vesicular, aphyric/microcrystalline	Type-C	Plg, Cpx, Ox, Ol, Opx, Amph		50	40	10				
**NIS 426**	Cape Katzouni	36°36′55.38″N	27°11′25.52″E	18	8	Lag-breccia	Crystal rich clast	ca. 15 cm	vesicular, microcrystalline	Type-C	Plg, Cpx, Ox, Ol, Opx, Amph	55/45	45	40	15	✓		✓	10
**NIS 427**	Cape Katzouni	36°36′55.38″N	27°11′25.52″E	18	8	Lag-breccia	Crystal rich clast	ca. 40 cm	vesicular, microcrystalline	Type-C	Plg, Cpx, Ox, Ol, Opx, Amph	75/25	43	45	12	✓		✓	5
**NIS 428**	Cape Katzouni	36°36′55.38″N	27°11′25.52″E	18	8	Lag-breccia	Crystal rich clast	ca. 15 cm	high vesicular, microcrystalline	Type-C	Plg, Cpx, Ox, Ol, Opx, Amph	50/50	40	45	15				5
**NIS 429**	Cape Katzouni	36°36′55.38″N	27°11′25.52″E	18	8	Lag-breccia	Crystal rich clast	10–15 cm	low vesicular, aphyric	Type-B	Plg, Ox, Opx, Amph	60/40	45	35	20			✓	15
**NIS 430**	Cape Katzouni	36°36′55.38″N	27°11′25.52″E	18	8	Lag-breccia	Crystal rich clast	ca. 10 cm	low vesicular, aphyric/microcrystalline	Type-B	Plg, Cpx, Ox, Ol, Amph	60/40	45	40	15			✓	10
**NIS 431**	Nikia - main road	36°34′47.52″N	27°11′16.38″E	421	9	Diluted PDC	Pumice	ca. 20 cm	porphyritic		Plg, Cpx, Ox, Opx, Amph	95/5	7	45	48	✓	✓		
**NIS 433**	Nikia - main road	36°34′47.52″N	27°11′16.38″E	421	9	Diluted PDC	Pumice	ca. 40 cm	porphyritic		Plg, Cpx, Ox, Opx, Amph	95/5	5	50	45	✓	✓		
**NIS 434**	Nikia - main road	36°34′47.52″N	27°11′16.38″E	421	9	Diluted PDC	Crystal rich clast		vesicular, aphyric/microcrystalline	Type-B	Plg, Cpx, Ox, Ol, Opx, Amph	85/15	35	55	10		✓	✓	2
**NIS 435**	Nikia - main road	36°34′47.52″N	27°11′16.38″E	421	9	Diluted PDC	Crystal rich clast	6–8 cm	low vesicular, aphyric/microcrystalline	Type-B	Plg, Cpx, Ox, Ol, Opx, Amph	80/20	30	55	15		✓		2
**NIS 436c**	Nikia - main road	36°34′47.52″N	27°11′16.38″E	421	9	Diluted PDC	Crystal rich clast	4–5 cm	vesicular, aphyric/microcrystalline	Type-B	Plg, Cpx, Ox, Ol, Opx, Amph	70/30	40	53	7		✓	✓	
**NIS 436d**	Nikia - main road	36°34′47.52″N	27°11′16.38″E	421	9	Diluted PDC	Crystal rich clast		low vesicular, aphyric/microcrystalline	Type-A	Plg, Cpx, Ox, Ol, Opx, Amph	90/10	35	50	15		✓	✓	2

*Samples with mingling features are doubled to describe crystal-rich portions and pumiceous portions; "-" thin secrtion not available

CRC: Crystal-rich Clast; Plg: plagioclase; Cpx: clinopyroxene; Opx: orthopyroxene; Amph: amphibole; Ol: olivine; Ox: oxides; ph: phenocrysts (crystals >0.5 mm); mph: micro-phenocrysts (crystals >0.3 mm). Reaction rims: presence of olivines and/or opx with reaction rims to amphiboles; ph+mph in CRC (%): estimated abundance of crystals coarser than the average size of the microcrystalline groundmass in the CRCs.

**Fig. 2:**
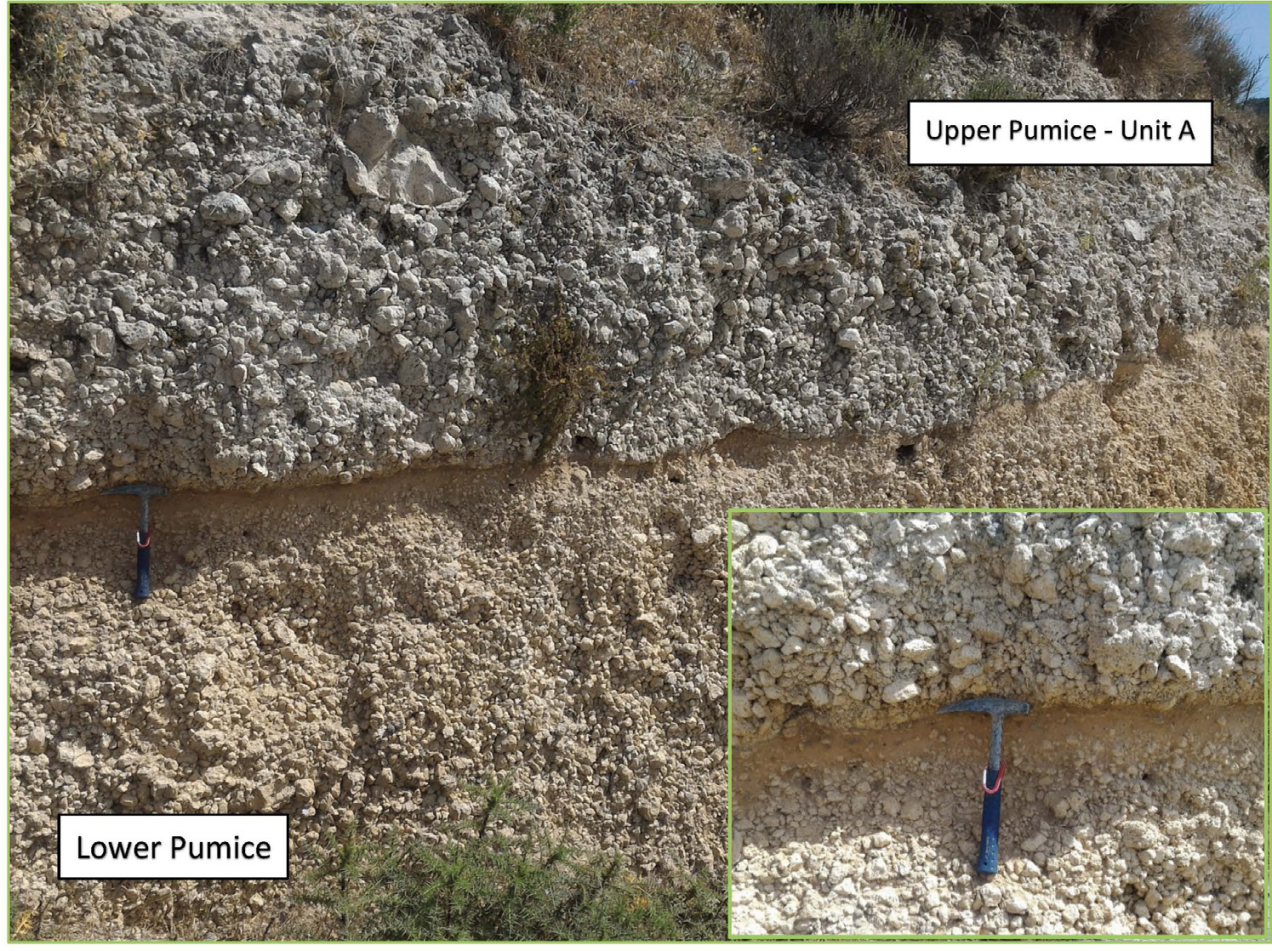
Images of the paleosoil horizon marking the base of the UP pyroclastic sequence (a detail is showed in the inset) and the contact with the deposit of the previous Lower Pumice (LP) explosive eruption.

**Fig. 3:**
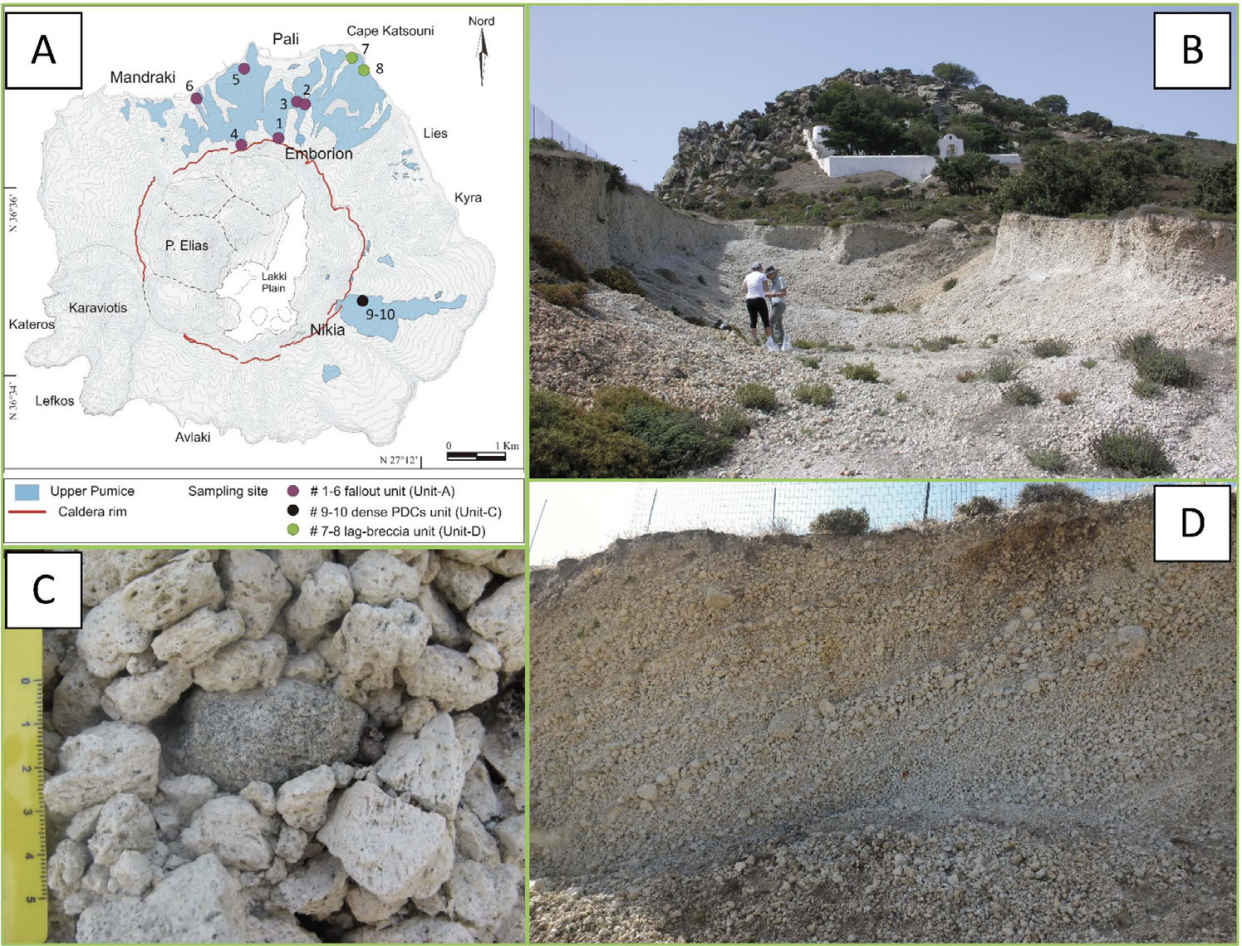
Selection of representative images of the basal fallout unit (Unit-A) outcropping in the northern part of the caldera rim (outcrop 1) near the Emborion village. (A) schematic map of the UP distribution together with the location of the different sampling site (see legend for detail); (B) main view of the outcrop 1. The Unit-A consists in a 0.5 to 8 m thick level of unconsolidated, granular sustained, moderately assorted, massive fallout, mainly composed of white sub-angular pumices, with size varying from lapilli to small blocks and dense Crystal-rich Clasts (CRC) (C). Occasional evident stratification, formed by layers of clasts at different grain size, is observed (D) and interpreted as the result of syn-depositional reworking in the most proximal deposits [2], [3], [4].

**Fig. 4:**
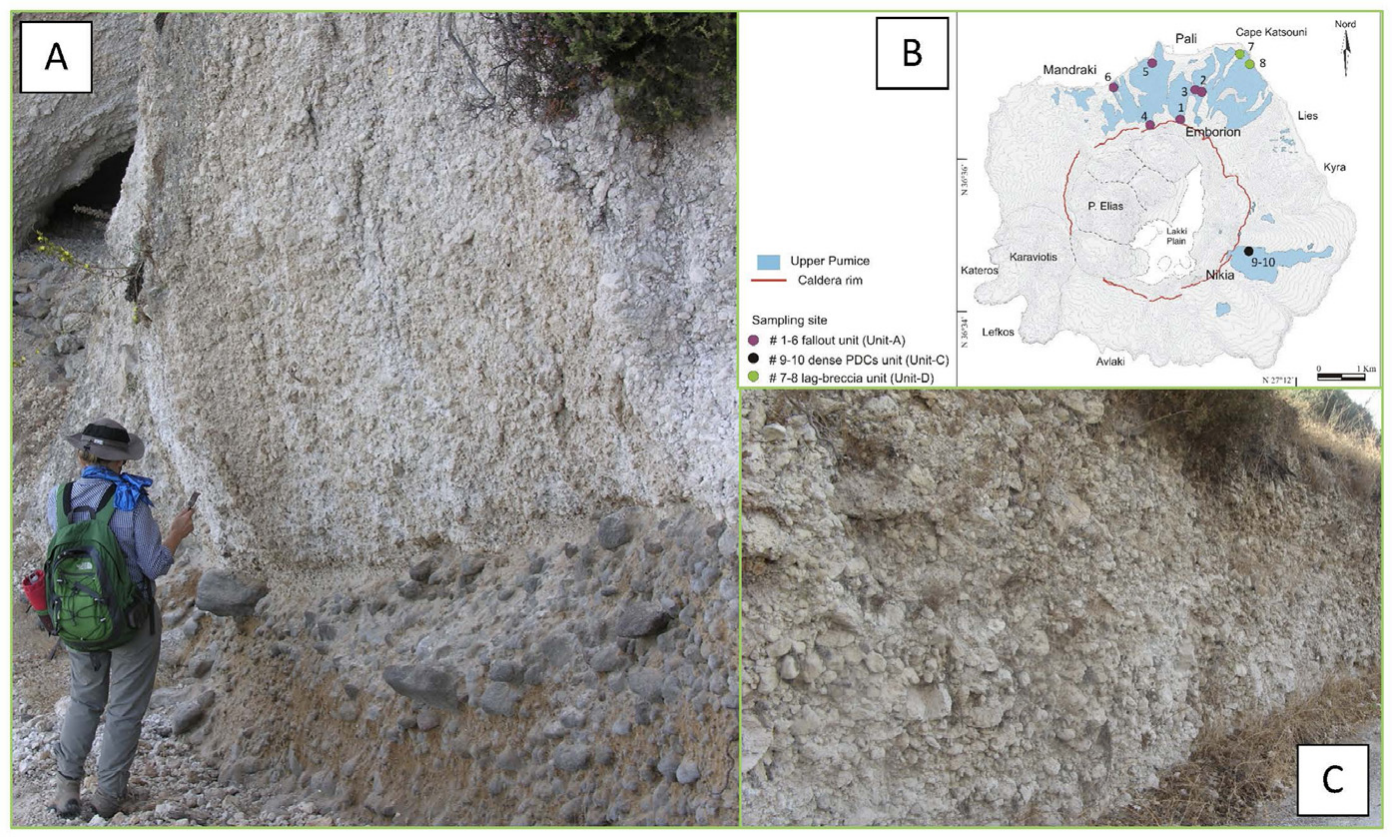
Representative images of the basal fallout unit (Unit-A) outcropping in the northern part of the caldera, just inside the rim border (outcrop 4) (A) and along the main road, near the coast above Pali village (outcrop 5) (C). Pumices are the prevalent juvenile components whereas CRCs constitute about 5% of the deposit. The lithic content is less than 2%, there are very small quantities of fine ash and loose crystals as matrix. This unit has been interpreted as pyroclastic fall deposit, emplaced from the column of a Plinian or sub-Plinian eruption [2,^3^,5]. The schematic map of the UP distribution and outcrops location is also reported (B).

**Fig. 5:**
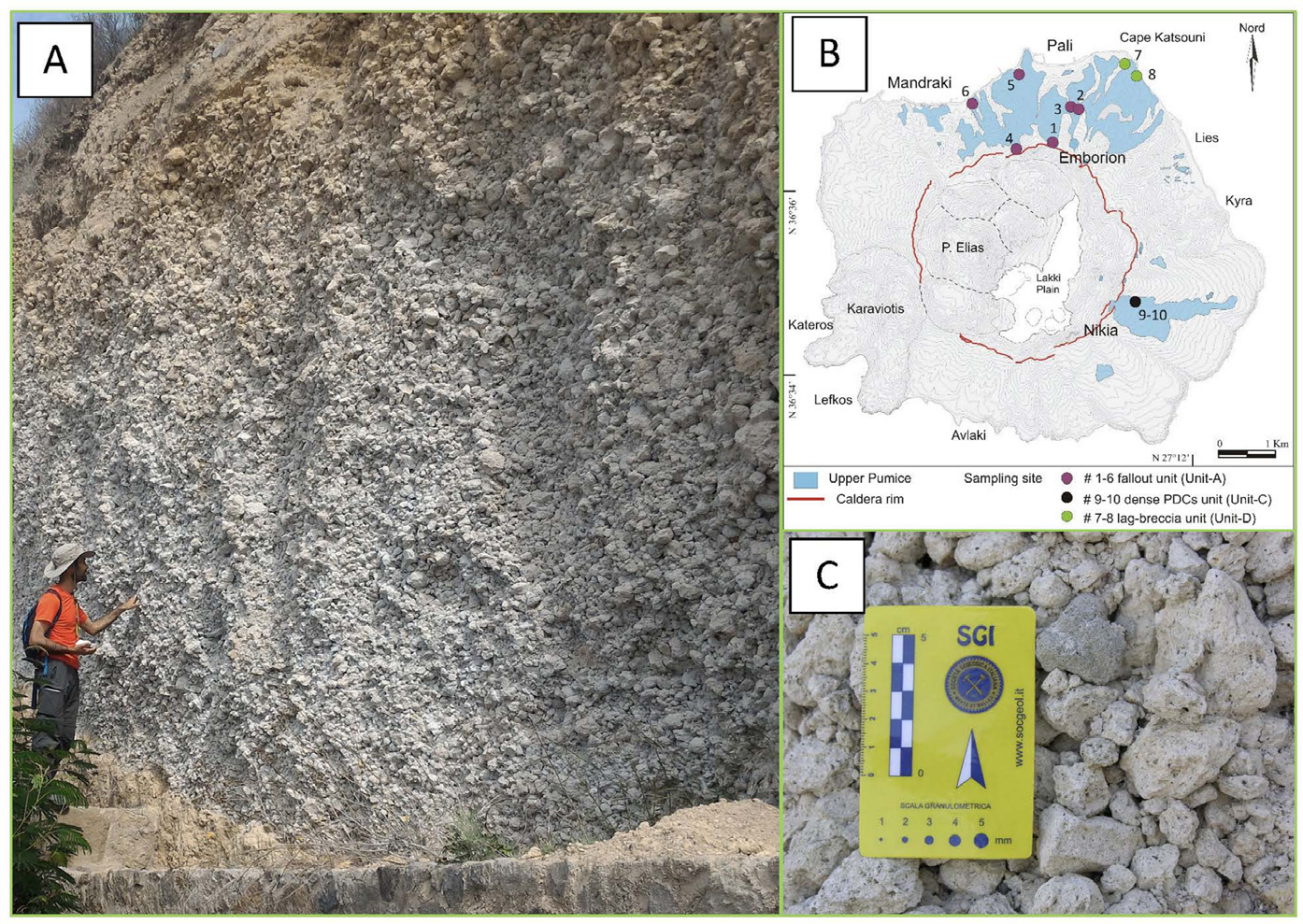
Representative image of outcrop 6 where the fallout unit is particularly well preserved (A). CRCs clasts are evident within the pumices showing grey colour and globular shapes (C). The schematic map of the UP distribution and outcrops location is also reported (B) (For interpretation of the references to colour in this figure legend, the reader is referred to the web version of this article).

**Fig. 6:**
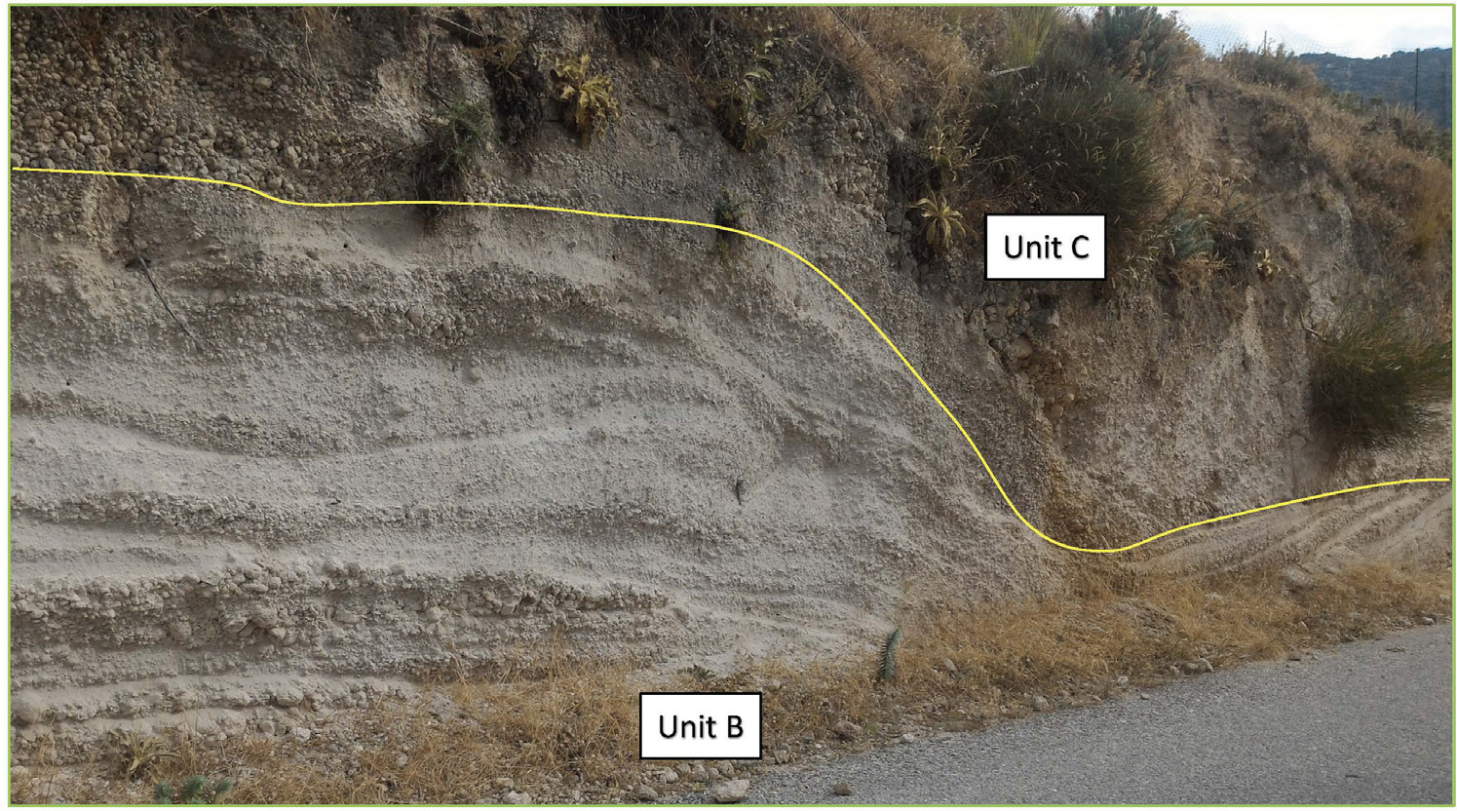
Representative image of the second unit (Unit-B) at the contact with the overlying Unit-C, close to outcrop 7, along the main road to Cape Katsouni, in the north-east part of Nisyros. Unit-B is a succession of several layers of fully diluted pyroclastic current (according to [6]) alternated with fallout levels. The flow levels are formed by a matrix of ash and loose crystals where sub- to well-rounded pumice lapilli are immersed, alternating with layers of coarser ash. Unit-C is a massive deposit of unconsolidated material, composed of coarse ash, fine lapilli and loose crystals, with well rounded, slightly vesiculated pumice and dispersed lithic clasts [2], interpreted as a granular fluid-based current (according to [6]).

**Fig. 7:**
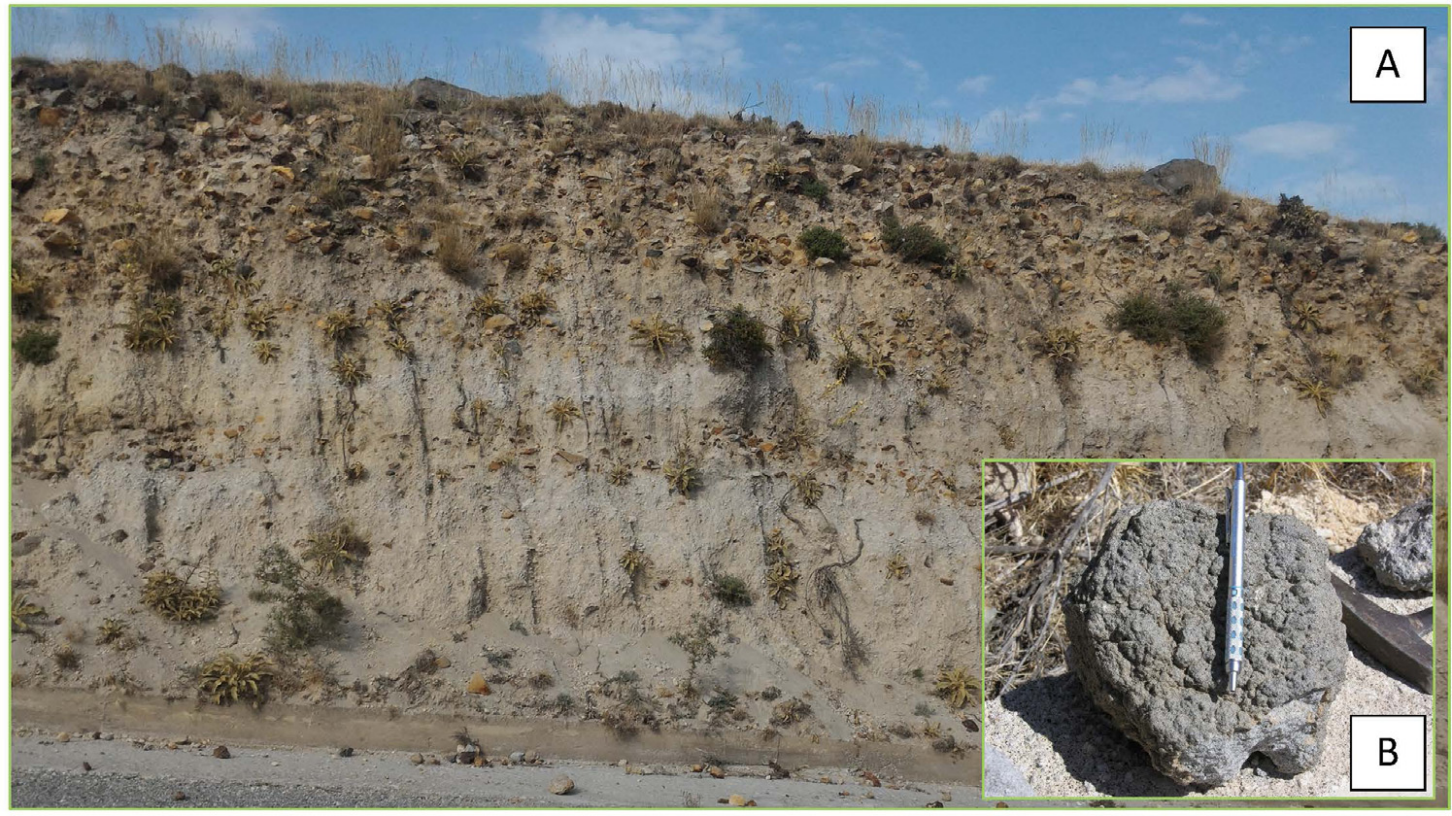
Representative image of the principal outcrop of unit-D exposed on the main street south of Cape Katsouni (outcrop 8). Unit-D is a dense pyroclastic current, gradually interlayered toward the top with lithic-rich lenses. This unit is constituted by a breccia deposit composed of rounded pumices and abundant (up to 15%) dense juvenile clasts with crenulated or "bread crust" surfaces, up to few tens of centimetres in diameter, and angular lithic clasts within an unconsolidated ash matrix including. Lithics mainly consist of fresh and hydrothermalised lava clasts; fragments of hypoabyssal igneous rocks, skarn and limestone with hydrothermal alteration are also present [as also reported by 2, 3]. Unit-D is interpreted as a lag-breccia deposit [2], emplaced from a dense PDC formed by the collapse of the eruptive column as a consequence of the caldera collapse [7].

**Fig. 8:**
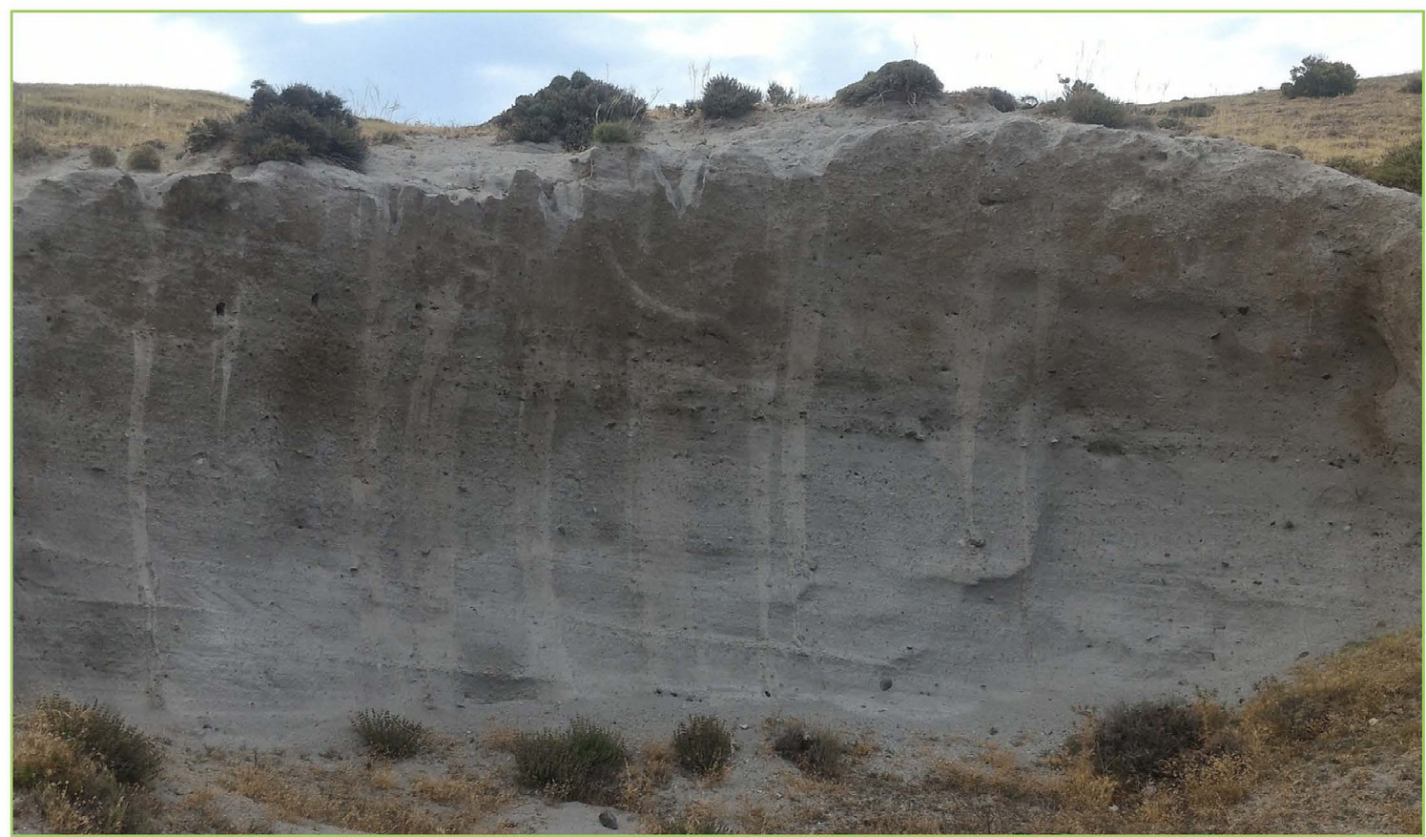
Image of the top unit of the UP sequence (Unit-E) composed by a massive or weakly laminated deposit formed by grey ash with loose crystals, rounded centimetre-sized pumice and lithic lava and limestone clasts (about 20%, [2]). This unit have been interpreted as a deposit from diluted pyroclastic density currents [2] or due to a phreatomagmatic eruptive event [5].

Representative photos of the sampled outcrops of the UP deposit are shown in Fig. 2, Fig. 3, Fig. 4, Fig. 5, Fig. 6, Fig. 7, Fig. 8 and illustrate in detail the different depositional units emplaced by the UP eruption and their juvenile components.

**Fig. 9:**
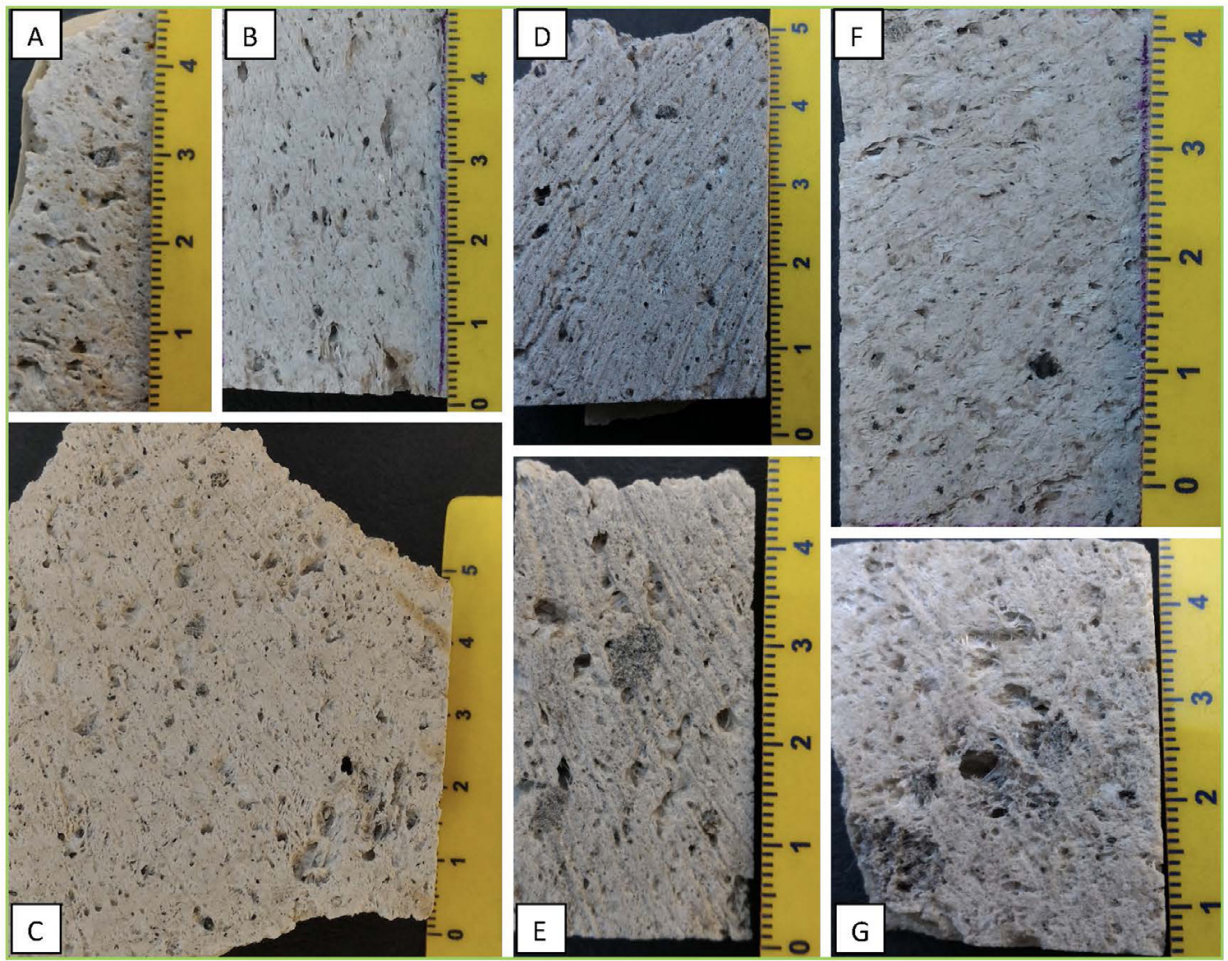
Cut blocks of pumice samples collected in different outcrops from the fallout deposit (A: NIS353; B: NIS374; C: NIS377) and from the lag-breccia deposit (D: NIS418; E: NIS419, outcrop 8). Pumices from the PDC deposits collected in outcrop 9 are also shown (F: NIS431; G: NIS433). Pumice clasts are white or pale yellow in colour, porphyritic and highly vesiculated, sub-angular, and range from 10 to 40 cm in diameter. Pumices often include micro-enclaves or grey bands (E, F) (For interpretation of the references to colour in this figure legend, the reader is referred to the web version of this article).

**Fig. 10:**
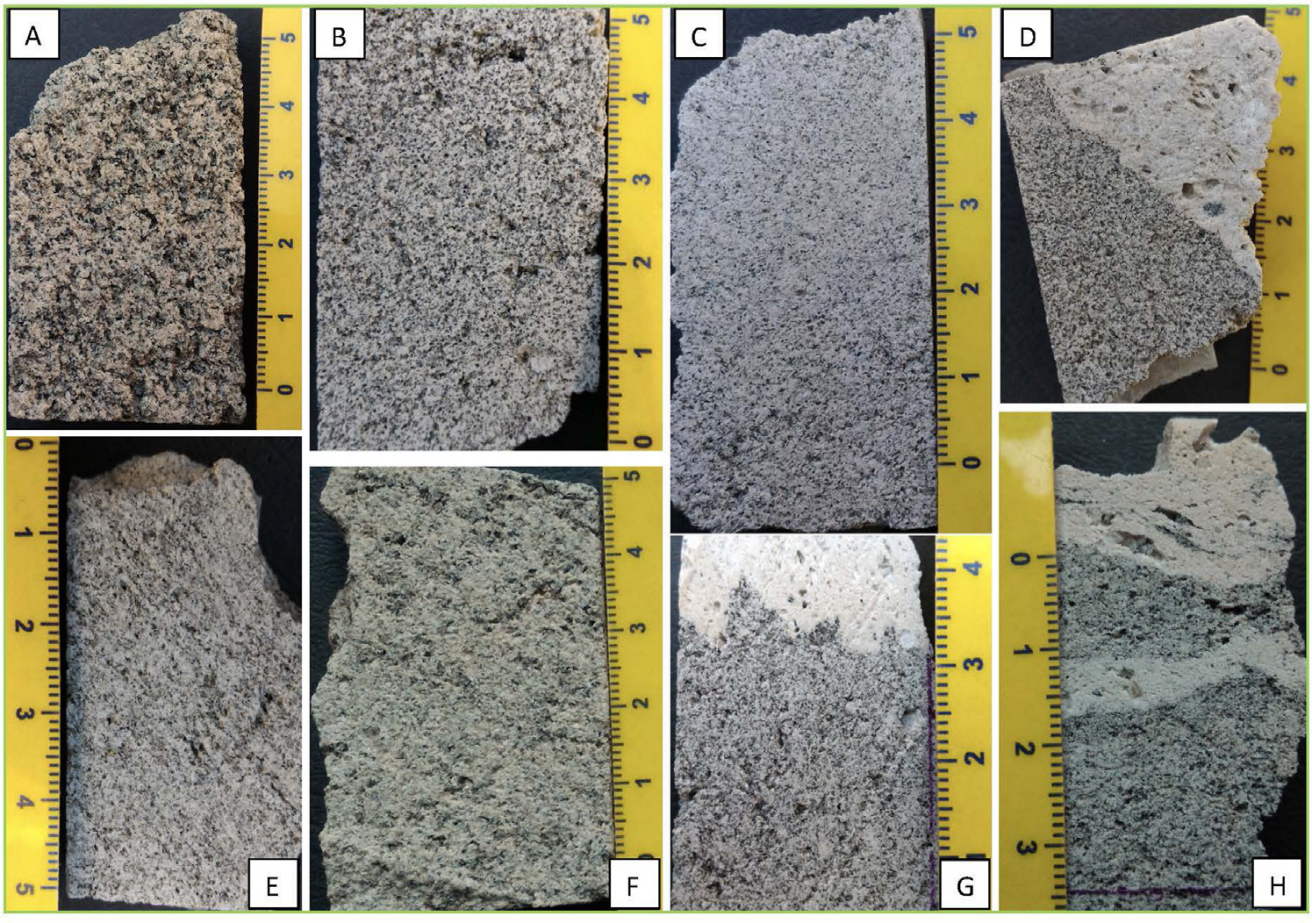
Cut blocks of representative CRC samples of the collected from the fallout deposits. A: NIS356; B: NIS357; C: NIS359; D: NIS358; E: NIS370; F: NIS371; G: NIS378; H: NIS381. See Table 1 for details.

**Fig. 11:**
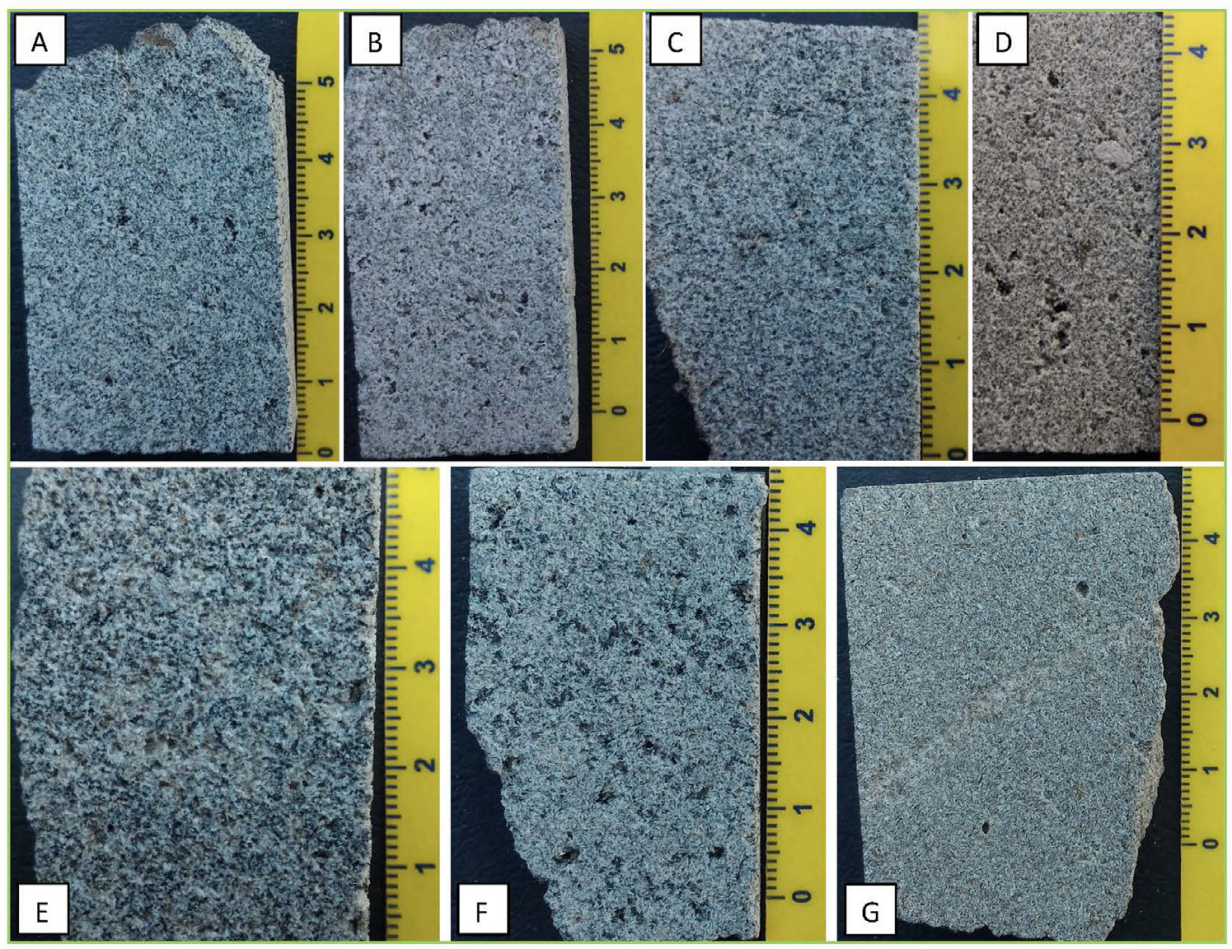
Cut blocks of representative CRC samples collected from the lag-breccia deposits (outcrop 8). A: NIS426; B: NIS425; C: NIS423; D: NIS416; E: NIS424; F: NIS427; G: NIS421. See Table 1 for details.

**Fig. 12:**
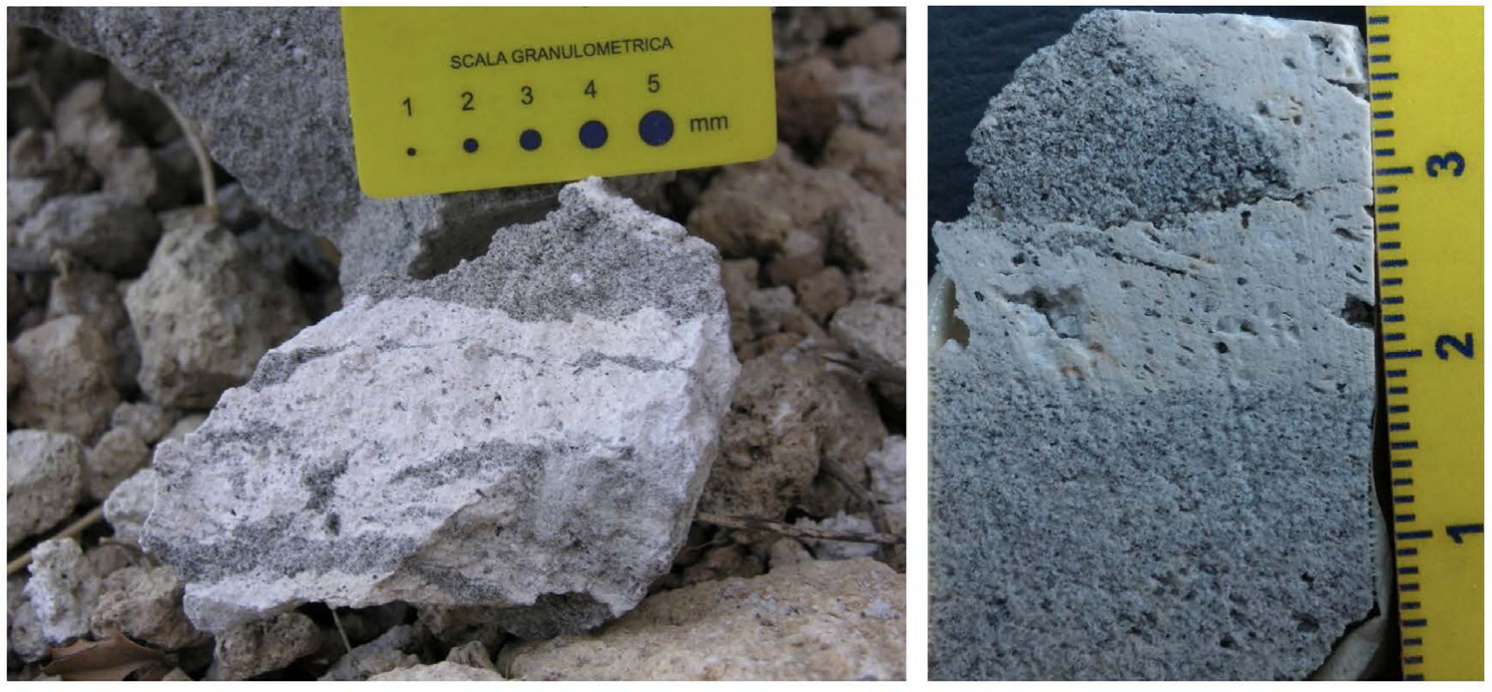
Representative photos of two samples showing the contacts between pumice and CRCs. The contacte between the two lithologies are sharp but convoluted due to a process of plastic interaction.

Fig. 9, Fig. 10, Fig. 11, Fig. 12 report selected representative images of cut blocks of samples and hand-specimen highlighting the difference between the two main lithotypes (pumice and crystal-rich clasts, hereafter CRCs) and within the CRC population itself. The CRCs show wide variation in their vesiculation and colour; the latter is due to the different proportion of crystal content (both for phases and size) and groundmass, varying from grey to white.

### 1.2 Petrography

In the next section, a detailed selection of microphotographs and backscattered (BSE) images (Fig. 13, Fig. 14, Fig. 15, Fig. 16, Fig. 17, Fig. 18, Fig. 19, Fig. 20, Fig. 21, Fig. 22) are reported to show the main petrographic characteristics of the studied samples (pumices and CRCs).

**Fig. 13:**
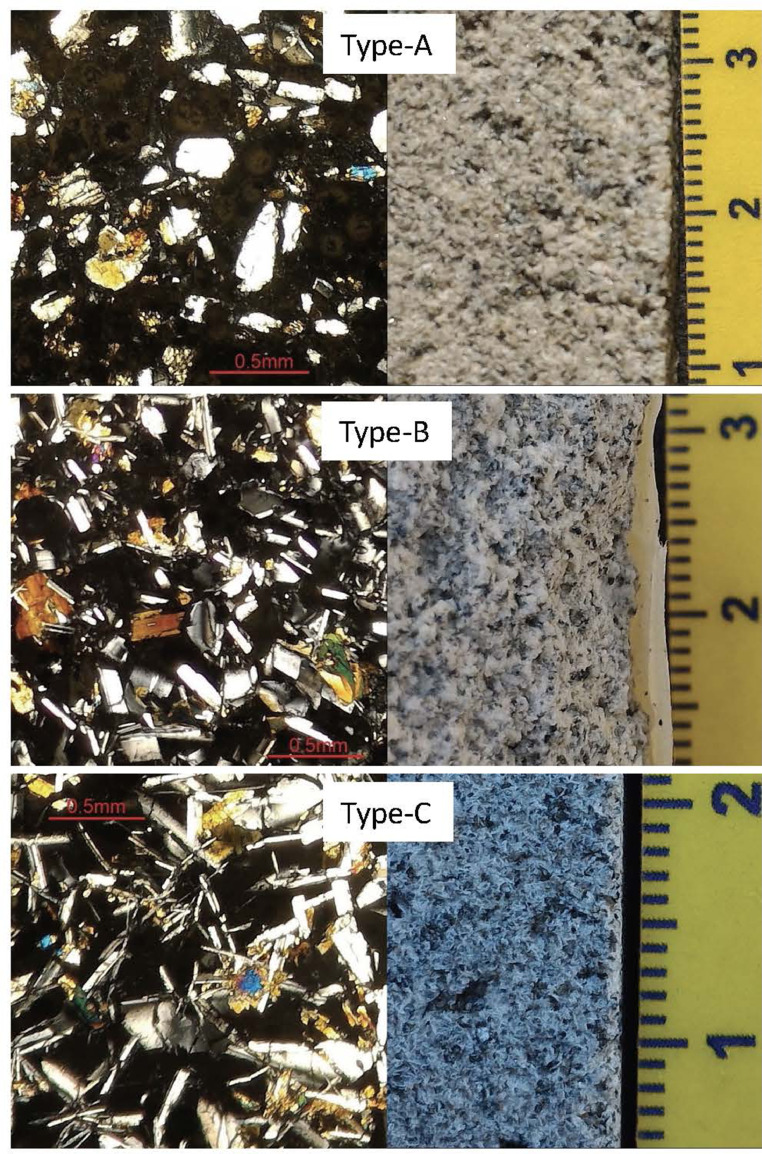
Comparison between hand-specimen blocks and the relative microphotographs acquired on the thin section, showing the three different textures types defined among the CRC samples.

**Fig. 14:**
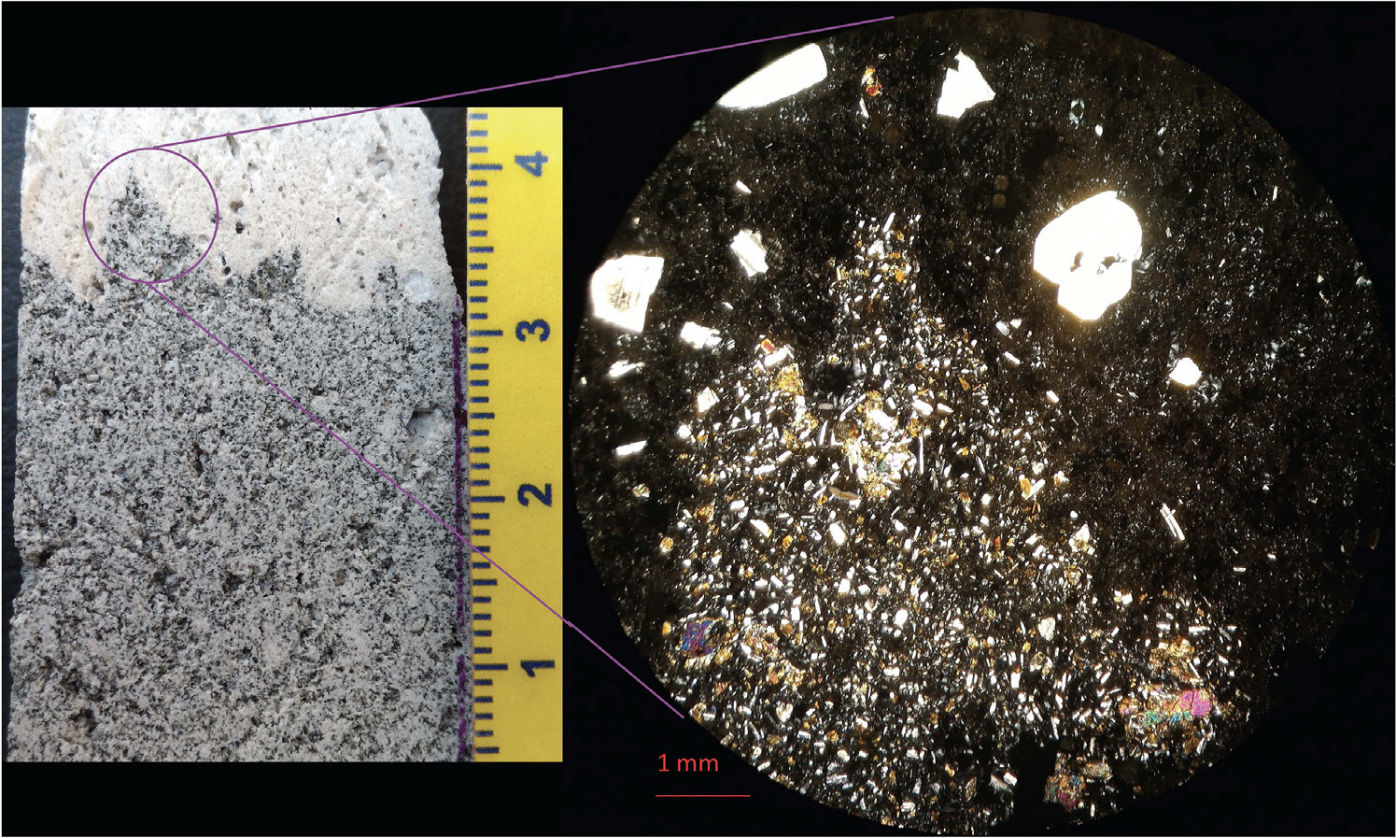
Cut block sample and the relative microphotograph detail of the contact between pumices and a dense crystal-rich clast. The dispersion of CRC portions into the pumice is evident both as loose crystals and micro-encalves.

**Fig. 15:**
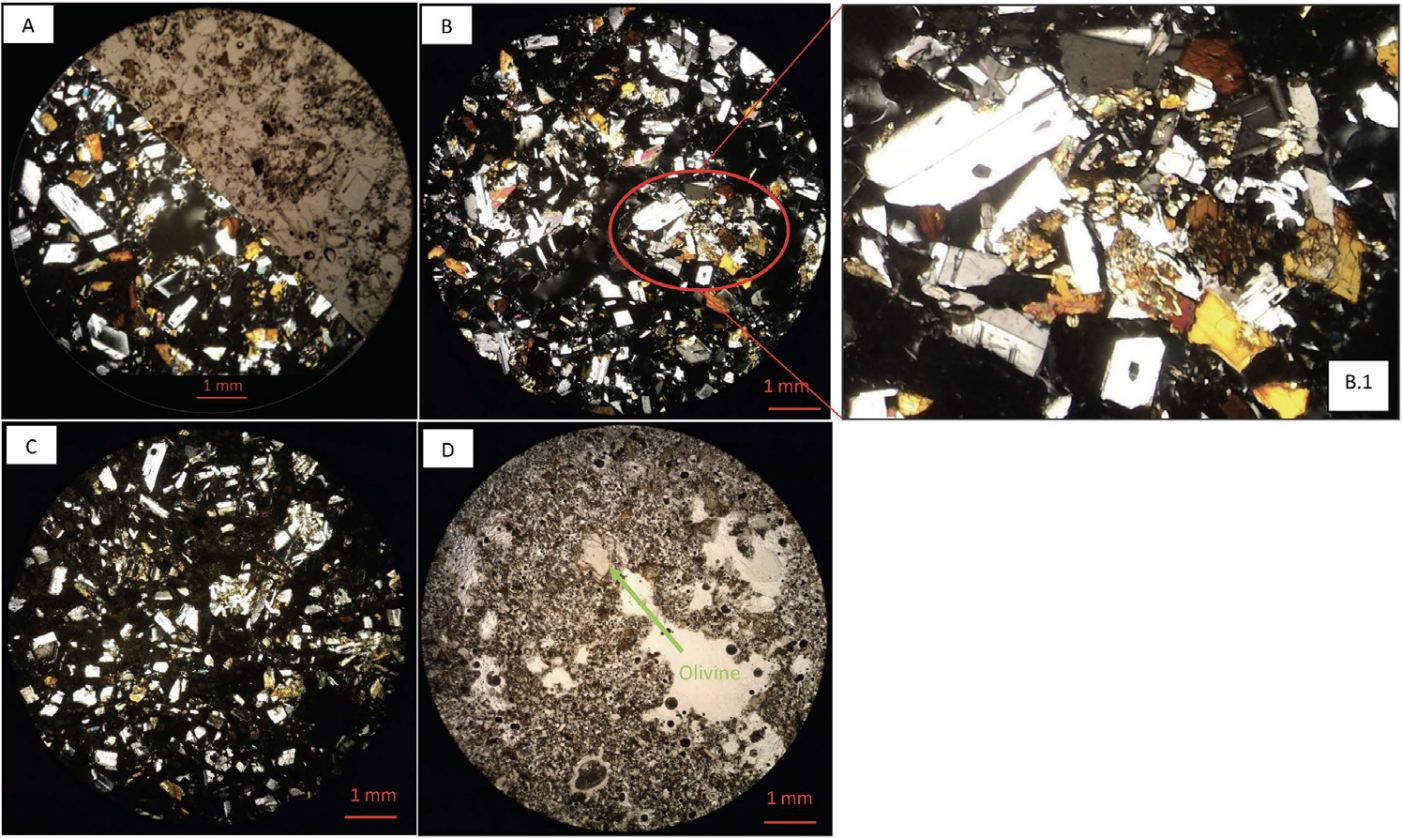
Thin section microphotograph of Type-A CRCs. A: NIS356, parallel and crossed nicols; B: NIS 356, detail of crystal cloth (B.1); C: NIS 371; D: NIS 378 with a resorbed olivine phenocryst (parallel nicols).

**Fig. 16:**
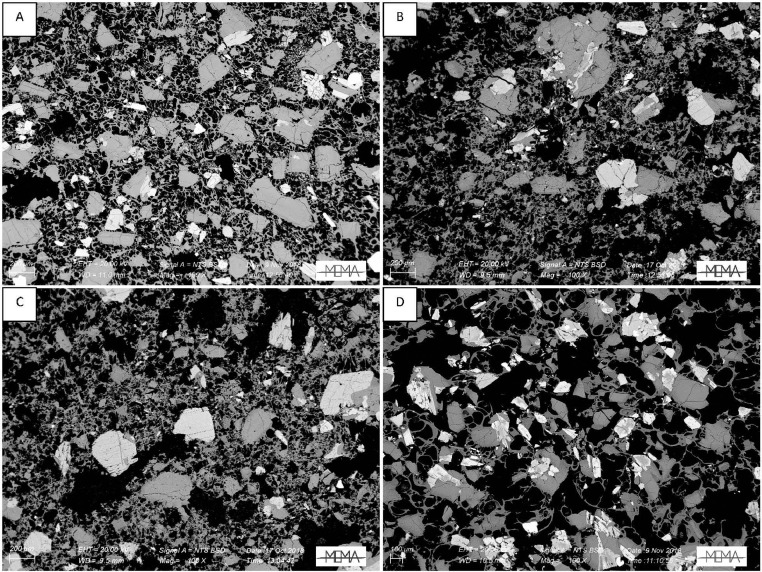
BSE images of Type-A CRC obtained by SEM. A: NIS317; B and C: NIS368d; D: NIS420.

**Fig. 17:**
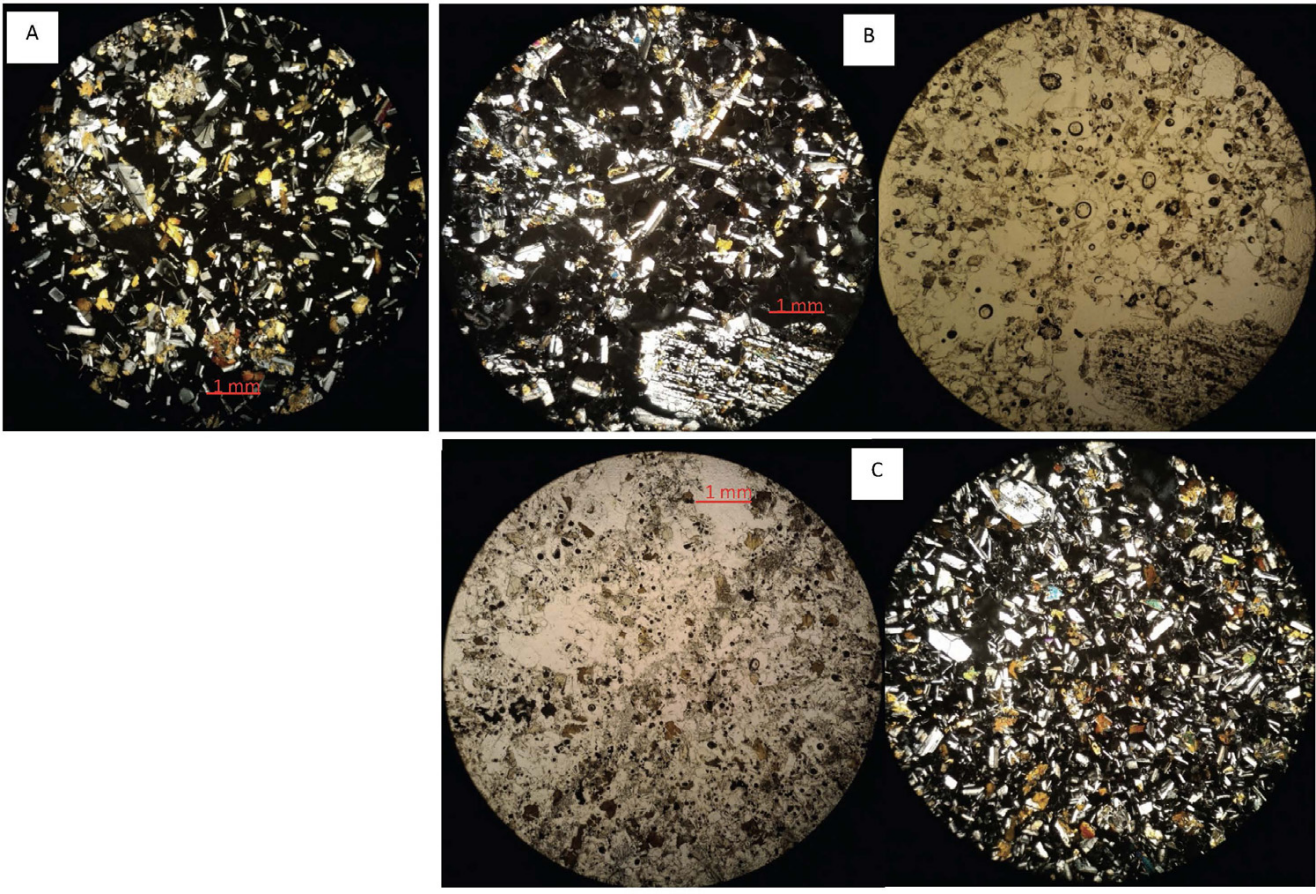
Thin section microphotograph of Type-B CRCs. A: NIS314, crystal-cloth presence; B: NIS423, parallel and crossed nicols, plagioclase phenocryst with sieved texture; C: NIS360, parallel and crossed nicols.

**Fig. 18:**
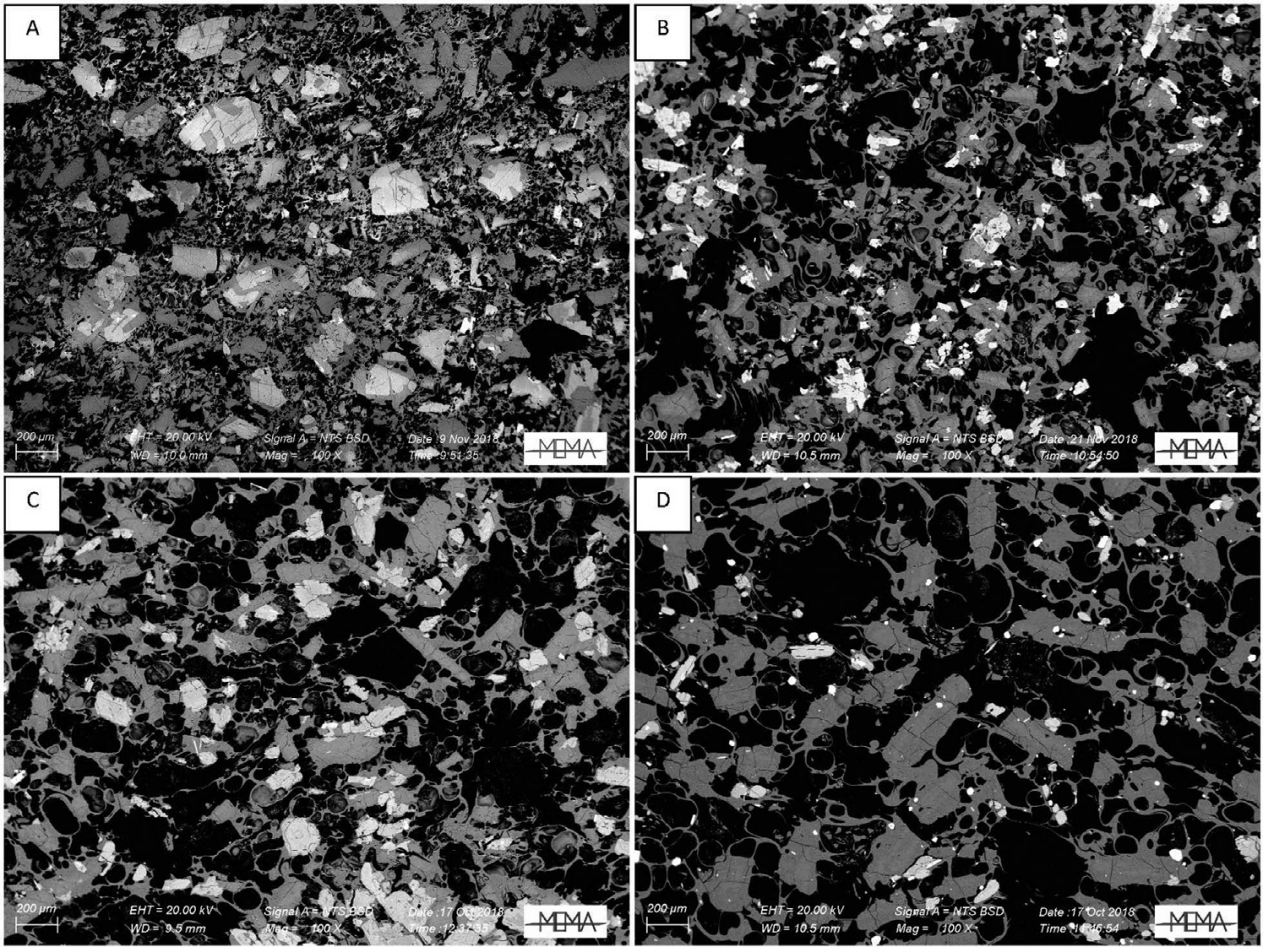
BSE images of Type-B CRCs from the fallout deposit obtained by SEM observation. A: NIS357; B: NIS 368b; C: NIS364; D: NIS318.

**Fig. 19:**
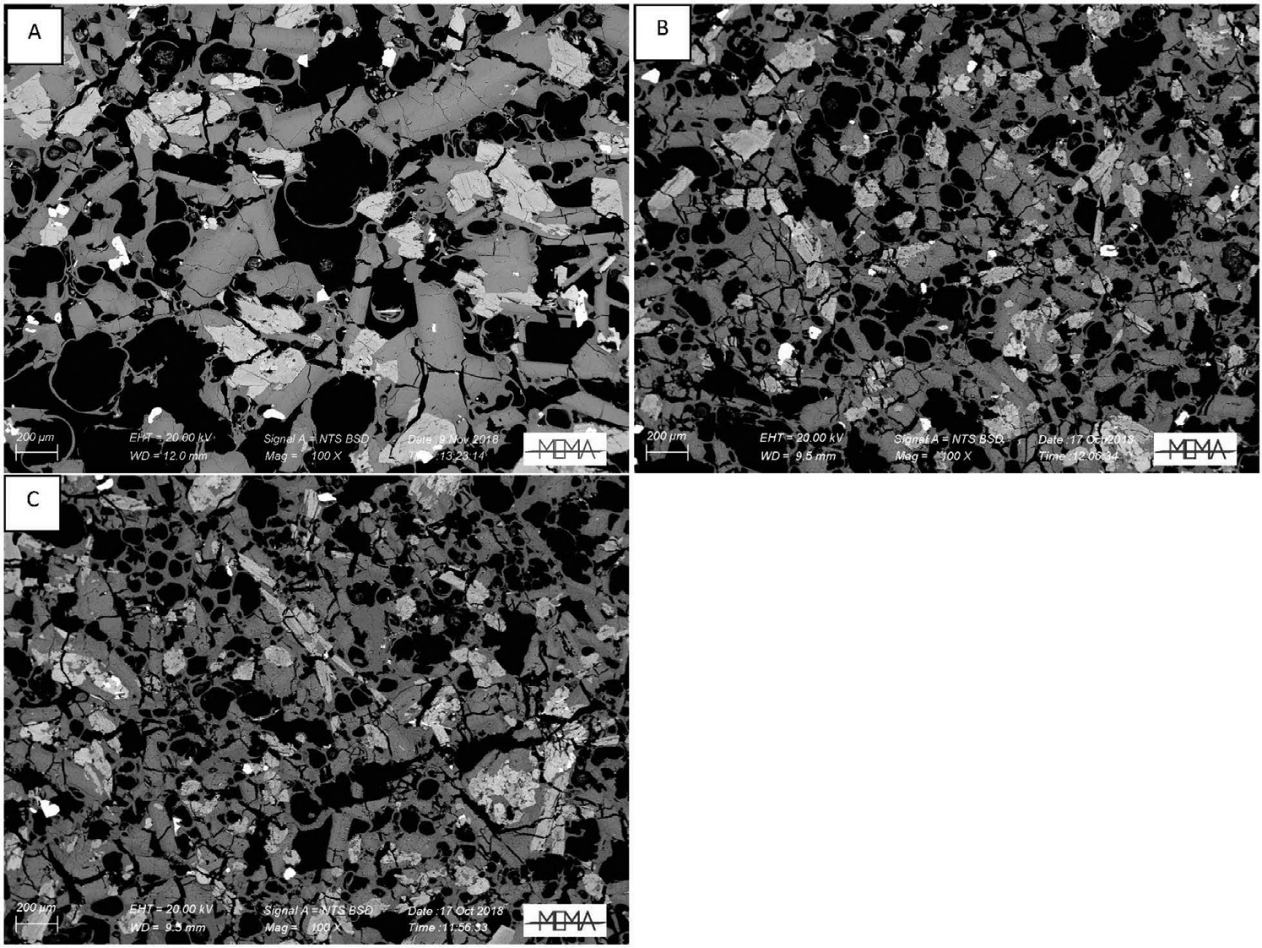
BSE images of Type-B CRCs from lag-breccia deposit obtained by SEM observation. A: NIS424; B and C: NIS 430.

**Fig. 20:**
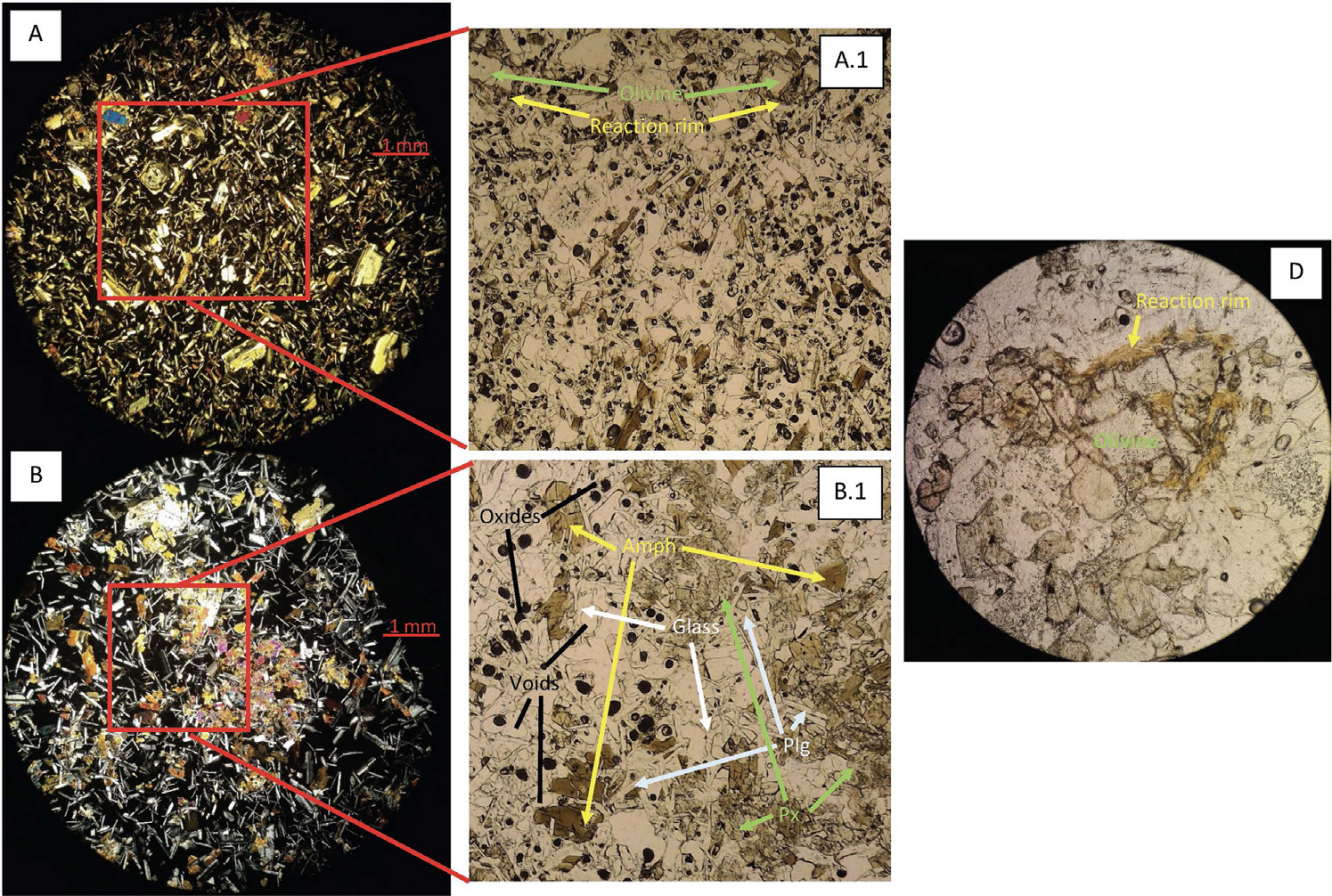
Thin section microphotograph of Type-C CRCs. A: NIS422, parallel nicols detail of diktytaxitic texture and olivine with reaction rim (A.1); B: NIS421, parallel nicols detail of diktytaxitic texture and crystal cloths (B.1). D: detail of olivine with reaction rim.

**Fig. 21:**
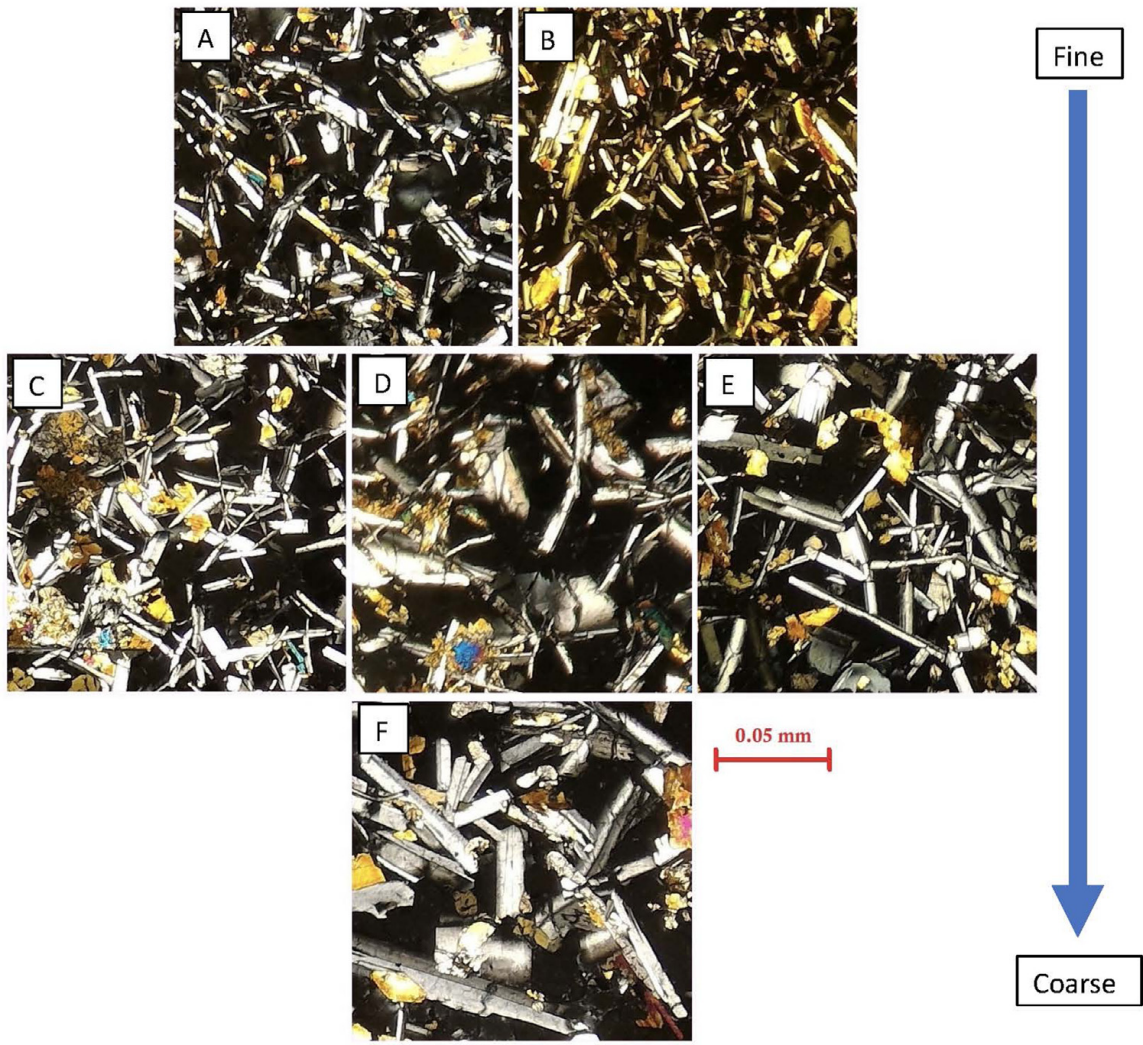
Examples of the grain size variations within the Type-C samples. From the top: A, NIS421, B, NIS422; middle: C, NIS426, D, NIS427, E, NIS313; bottom: F, NIS312.

**Fig. 22:**
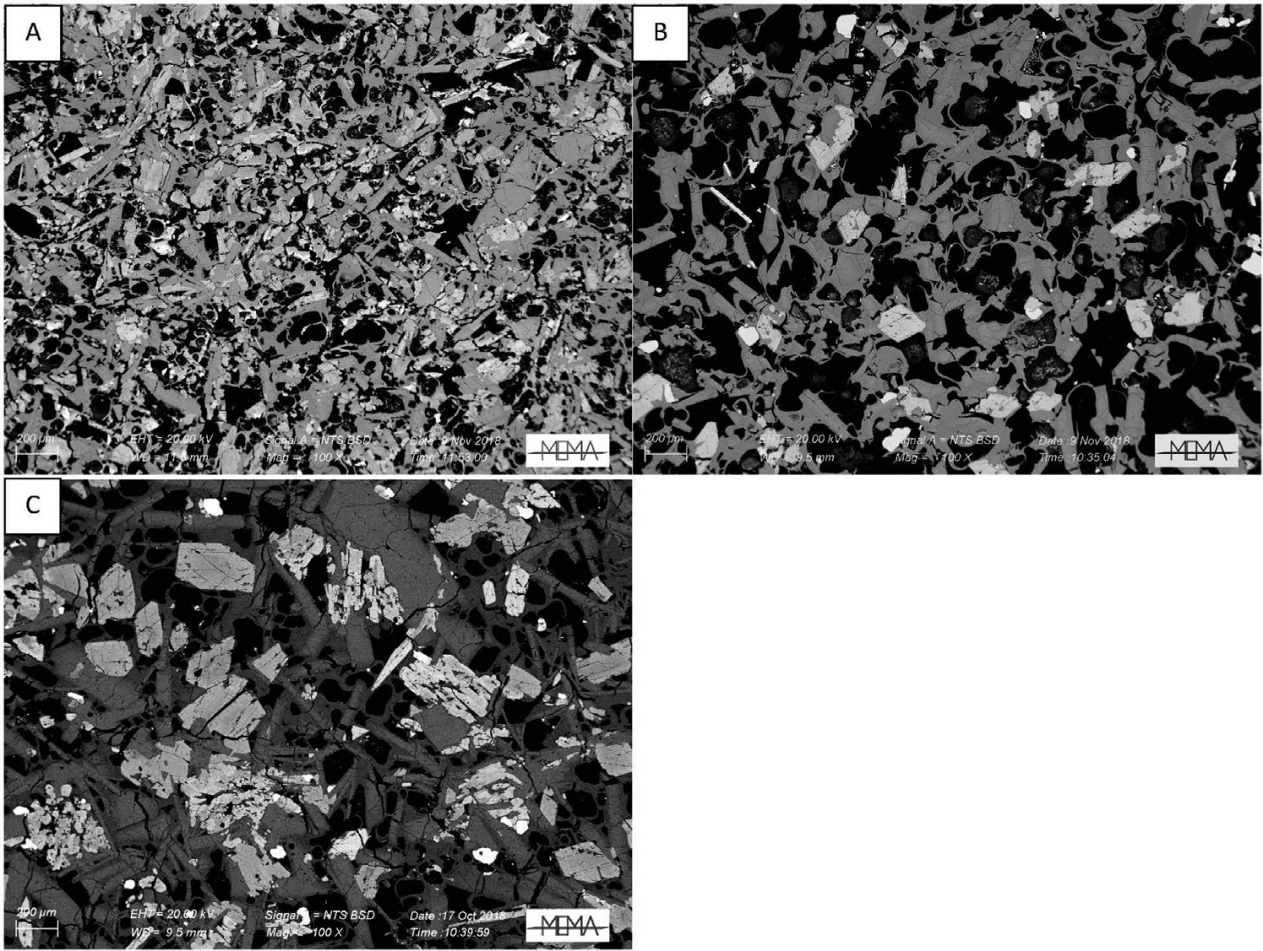
BSE images of Type-C CRC samples obtained by SEM observation. A: NIS379b; B: NIS422; C: NIS428.

Pumices from all the deposits have similar features. They are porphyritic, mainly composed by a glassy matrix and a crystal content up to 5–10%; vesicularity vary between 30% and 50%; the matrix appears often fibrous or fluidal. Paragenesis is mainly composed by plagioclase (always more than 75%), orthopyroxene and amphibole; clinopyroxene and olivine are rare; accessory phases are oxides and apatite, often included in orthopyroxene. Crystals are often found as glomeroporphyritic aggregates. Plagioclase phenocrysts sometimes show disequilibrium textures, with sieved cores, resorbed zones, resorbed or overgrowth rims.

Crystal-rich clasts are highly heterogeneous in textures and were subdivided in three groups, named Type-A, Type-B and Type-C. The paragenesis is similar between the three groups: plagioclase represents more than 50% of the mineral assemblage, followed by amphibole and pyroxenes; olivine is rare; accessories phases are oxides and apatite. Amphibole can be found associated with plagioclase to form the microcrystalline groundmass network, either with acicular or tabular habitus, or as reaction rims on pyroxenes. Rare amphibole and pyroxene phenocrysts can be up to 2 mm, while plagioclase phenocrysts can reach 6 mm.

Type-A clasts are mostly found in fallout deposits and are characterised by microcrystalline texture with almost equigranular crystal size (0.1–0.5 mm), constituted by tabular plagioclases, amphiboles and pyroxenes (mainly orthopyroxene), with variable oxides content. Crystals are dispersed in a glassy, highly vesiculated groundmass, without a defined fabric.

Type-B clasts are the more variable in terms of crystal content and size; they show microcrystalline, inequigranular, low porphyritic texture, with variable crystal orientation defining at places a sort of network, likely the Type-C textures. They are present both in the fallout and lag-breccia deposits.

Type-C clasts are mostly found in the lag-breccia deposits, and they are characterized by a equigranular, low porphyritic textures with diktytaxitic voids, formed by a network of acicular plagioclases and amphiboles, with interstitial pyroxene. They show interstitial glass and variable vesicle abundance, generally lower than the other two types.

### 1.3 Geochemistry

The following table (Table 2) reports the complete dataset of major and trace element data on 31 whole-rock samples of pumice and CRCs of the Upper Pumice activity. Selected incompatible trace elements and Rare Earth Elements (REE) together with Sr-Nd isotope ratios were also determined on a further selection of 22 samples.

**Table 2:** Major and trace element composition and Sr-Nd isotopic ratios of the studied samples from the Upper Pumice deposit (Nisyros, Greece).

Outcrop	8	8	8	5	5	5	5	1	1	1	1	1	1	2	2	3
Depositional unit	Lag-breccia	Lag-breccia	Lag-breccia	Fallout	Fallout	Fallout	Fallout	Fallout (U-A)	Fallout	Fallout	Fallout	Fallout	Fallout	Fallout	Fallout	Fallout
Lithology	CRC	CRC	CRC	Pumice	CRC	CRC	CRC	Pumice	CRC	CRC	CRC	CRC	CRC	CRC	CRC	Pumice
Texture	Type-C	Type-C	Type-B	Porphyritic	Type-A	Type-A	Type-A	Porphyritic	Type-A	Type-B	Type-A	Type-A	Type-B	Type-A	Type-A	Porphyritic
Sample	NIS312*	NIS313*	NIS314*	NIS315*	NIS316a*	NIS316b*	NIS316c*	NIS 353	NIS 356	NIS 357	NIS 358	NIS 359	NIS 360	NIS 370	NIS 371	NIS 374

Major Elements wt% (water free)																
SiO_2_	56.74	56.45	57.33	71.43	62.04	60.15	64.17	70.58	57.87	58.17	60.69	62.72	57.61	64.14	59.27	71.66
TiO_2_	0.61	0.71	0.69	0.31	0.57	0.65	0.73	0.36	0.93	0.67	0.69	0.76	0.64	0.84	0.61	0.33
Al_2_O_3_	19.22	19.30	18.41	14.41	16.71	18.27	16.78	15.13	18.10	18.32	17.95	16.90	18.43	16.41	17.03	14.28
FeO*	5.64	5.86	5.59	2.35	4.73	5.72	4.50	2.49	5.87	5.50	5.01	5.01	5.35	4.76	5.18	2.42
MnO	0.10	0.10	0.11	0.06	0.09	0.10	0.08	0.07	0.11	0.10	0.09	0.10	0.10	0.09	0.09	0.07
MgO	4.73	4.55	4.51	1.06	3.68	2.15	2.17	0.99	4.04	4.38	3.25	2.43	4.70	2.05	4.65	0.86
CaO	8.88	8.48	8.51	2.62	6.80	8.61	5.05	2.93	8.09	8.58	6.88	5.76	8.78	5.21	8.63	2.76
Na_2_O	2.86	3.30	3.34	4.51	3.39	2.68	4.18	4.13	3.37	2.88	3.54	3.88	2.89	4.05	2.97	4.27
K_2_O	1.12	1.12	1.38	3.20	1.89	1.55	2.16	3.23	1.48	1.31	1.82	2.25	1.40	2.29	1.46	3.26
P_2_O_5_	0.10	0.13	0.13	0.06	0.10	0.12	0.18	0.08	0.14	0.08	0.08	0.19	0.10	0.16	0.09	0.08
Total	100.00	100.00	100.00	100.00	100.00	100.00	100.00	100.00	100.00	100.00	100.00	100.00	100.00	100.00	100.00	100.00
LOI	0.58	0.78	0.71	3.05	1.83	1.34	1.77	3.25	1.60	1.63	2.08	2.07	1.66	2.21	1.58	2.74

Trace Elements (ppm)																
Be	nd	nd	nd	nd	nd	nd	nd	2	1	1	1	2	1	2	1	2
Sc	nd	nd	nd	nd	nd	nd	nd	5	20	19	15	11	19	12	19	4
V	*144*	143	*136*	31	107	*121*	*93*	39	167	146	136	90	140	98	130	35
Cr	*9.4*	5.8	*3.6*	3.4	18.0	*11.9*	*9.3*	bdl	bdl	bdl	bdl	bdl	bdl	bdl	20.0	bdl
Co	*22.4*	22.4	*20.6*	4.4	17.5	*19.7*	*10.5*	4	17	17	14	11	18	10	17	4
Ni	*3.29*	4.56	*4.19*	0.84	7.76	*5.85*	*1.27*	bdl	bdl	bdl	bdl	bdl	bdl	bdl	bdl	bdl
Cu	nd	bdl	nd	bdl	bdl	nd	nd	bdl	10	bdl	20	bdl	bdl	bdl	10	bdl
Zn	nd	bdl	nd	bdl	bdl	nd	nd	40	70	50	50	60	50	50	50	40
Ga	nd	13.8	nd	19	12.45	nd	nd	13	16	14	15	15	15	16	14	13
Rb	*34.6*	38.0	*42.2*	89.0	15.7	*45.5*	*38.8*	85	30	23	42	52	27	61	28	87
Sr	*571*	630	*606*	262	439	*552*	*379*	286	544	555	491	403	587	364	549	266
Y	*10.7*	13.7	*12.5*	19.0	18.1	*13.6*	*25.3*	17.2	22.9	16.4	17.4	20.7	15.7	29.2	16.3	16.5
Zr	*114*	123	*126*	188	143	*129*	*217*	219	157	128	153	189	122	227	139	194
Nb	*7.0*	7.6	*8.4*	12.6	8.5	*7.5*	*14.1*	10.4	9.2	5.6	7.1	9.4	6.1	11.8	5.8	9.2
Cs	bdl	0.6	bdl	4.0	1.0	bdl	bdl	2.8	1	0.6	1.2	1.4	0.7	1.8	0.8	2.9
Ba	*212*	258	*276*	710	348	*286*	*463*	786	315	253	391	429	258	484	291	775
La	*13.6*	17.8	*17.7*	38.4	17.0	*13.6*	*28.8*	40.4	22.3	14.5	18.5	24.3	14.9	38.1	16.4	35.7
Ce	*36.4*	31.5	*33.0*	53.4	28.8	*30.8*	*50.3*	65.2	40.6	28.5	37.2	46	29.5	56.7	33.8	59.5
Pr	nd	4.0	nd	6	4.3	nd	nd	7.06	5.26	3.43	3.99	5.29	3.53	8.42	3.81	5.83
Nd	*18.3*	13.9	*14.9*	21.9	12.6	*16.0*	*21.7*	22.8	20.1	13.5	15.1	19.5	13.6	31.7	14.5	19
Sm	nd	3.6	nd	3.2	3.5	nd	nd	3.72	4.36	3.03	3.13	4.09	3.03	6.26	3.02	3.03
Eu	nd	1.1	nd	0.7	0.9	nd	nd	0.76	1.21	0.91	0.89	1.06	0.94	1.32	0.86	0.71
Gd	nd	3.7	nd	3.00	3.13	nd	nd	3.10	4.31	2.97	2.94	3.80	2.96	5.33	2.98	2.68
Tb	nd	0.6	nd	0.4	0.5	nd	nd	0.48	0.67	0.48	0.49	0.58	0.47	0.9	0.49	0.43
Dy	nd	3.5	nd	2.5	3.0	nd	nd	2.83	4.01	2.86	2.99	3.47	2.89	5.19	2.91	2.59
Ho	nd	0.7	nd	0.49	0.61	nd	nd	0.57	0.81	0.57	0.6	0.73	0.55	1.01	0.55	0.52
Er	nd	1.9	nd	1.4	1.8	nd	nd	1.79	2.27	1.68	1.76	2.09	1.6	2.86	1.69	1.62
Tm	nd	0.27	nd	0.20	0.23	nd	nd	0.27	0.34	0.24	0.25	0.30	0.22	0.43	0.23	0.27
Yb	nd	1.9	nd	1.7	1.7	nd	nd	1.95	2.22	1.63	1.78	2.13	1.5	2.9	1.56	1.9
Lu	nd	0.30	nd	0.28	0.27	nd	nd	0.33	0.35	0.27	0.31	0.36	0.26	0.50	0.27	0.33
Hf	nd	3.6	nd	4.3	3.4	nd	nd	5.0	4.2	3.2	3.7	4.5	3.1	6.0	3.6	4.4
Ta	bdl	0.6	bdl	1.1	0.6	bdl	bdl	1.14	0.74	0.49	0.64	0.92	0.50	1.04	0.53	1.15
Pb	*6.0*	6.3	*6.9*	14.0	7.8	*11.6*	*11.0*	14	8	8	8	9	5	11	6	14
Th	*4.6*	2.7	*5.0*	13.0	8.6	*6.3*	*9.9*	11.9	4.42	3.19	4.76	5.94	3.13	7.52	3.96	11.4
U	nd	nd	nd	bdl	nd	nd	nd	3.44	1.17	0.86	1.27	1.6	0.84	1.94	1.04	3.31

Ratios																
Zr/Ba	0.54	0.48	0.46	0.26	0.41	0.45	0.47	0.28	0.50	0.51	0.39	0.44	0.47	0.47	0.48	0.25
Rb/Sr	0.06	0.06	0.07	0.34	0.04	0.08	0.10	0.30	0.06	0.04	0.09	0.13	0.05	0.17	0.05	0.33
Sr/Ba	2.69	2.45	2.20	0.37	1.26	1.93	0.82	0.36	1.73	2.19	1.26	0.94	2.28	0.75	1.89	0.34
La/SmN		3.47		8.40	3.42			7.60	3.58	3.35	4.14	4.16	3.44	4.26	3.80	8.25
Tb/YbN		1.23		0.96	1.22			1.00	1.23	1.20	1.12	1.11	1.28	1.27	1.28	0.92
Eu/Eu*		0.98		0.72	0.91			0.71	0.90	0.98	0.94	0.86	1.01	0.73	0.92	0.79

Isotope ratios																
^87^Sr/^86^Sr	0.704342	0.704255	0.704202	0.704563	0.704478	0.704327	0.704754	0.704532	0.704595	0.704396	0.704441	0.704580	0.704313	0.704876	nd	nd
2se	0.000005	0.000007	0.000006	0.000006	0.000006	0.000007	0.000006	0.000005	0.000004	0.000005	0.000006	0.000006	0.000005	0.000006	nd	nd
^143^Nd/^144^Nd	0.512533	0.512591	0.512616	0.512615	0.512552	0.512558	0.512556	0.512611	0.512539	0.512531	0.512560	0.512566	0.512537	0.512537	nd	nd
2se	0.000005	0.000005	0.000006	0.000005	0.000004	0.000004	0.000012	0.000009	0.000009	0.000008	0.000003	0.000009	0.000008	0.000010	nd	nd

Outcrop	4	4	4	6	7a	8	8	8	8	8	8	8	8	9	9	
Depositional unit	Fallout	Fallout	Fallout	Fallout	Lag-breccia	Lag-breccia	Lag-breccia	Lag-breccia	Lag-breccia	Lag-breccia	Lag-breccia	Lag-breccia	Lag-breccia	Diluted PDC	Diluted PDC	
Lithology	Pumice	CRC	CRC	Pumice	CRC	Pumice	Pumice	CRC	CRC	CRC	CRC	CRC	CRC	Pumice	Pumice	
Texture	Porphyritic	Type-A	Type-A	Porphyritic	Type-C	Porphyritic	Porphyritic	Type-C	Type-B	Type-B	Type-C	Type-C	Type-C	Porphyritic	Porphyritic	
Sample	NIS 377	NIS 378	NIS 381	NIS 401	NIS 416	NIS 418	NIS 419	NIS 421	NIS 423	NIS 424	NIS 425	NIS 426	NIS 427	NIS 431	NIS 433	

Major Elements wt% (water free)																
SiO_2_	69.05	60.84	59.05	71.11	58.99	71.22	71.27	59.63	58.65	56.65	57.42	57.17	57.44	70.97	71.04	
TiO_2_	0.36	0.63	1.01	0.35	0.66	0.34	0.34	0.66	0.72	0.75	0.65	0.72	0.64	0.34	0.34	
Al_2_O_3_	14.83	16.33	16.68	14.59	17.84	14.46	14.70	18.09	17.88	18.53	17.61	17.81	17.49	14.93	14.87	
FeO*	2.70	4.79	5.93	2.47	5.51	2.39	2.40	5.26	5.15	5.76	5.48	5.53	5.53	2.40	2.40	
MnO	0.07	0.09	0.11	0.07	0.10	0.07	0.07	0.11	0.10	0.11	0.10	0.10	0.10	0.07	0.07	
MgO	1.55	4.47	3.37	0.98	4.22	0.90	0.87	3.54	4.39	4.56	5.20	5.14	5.15	0.87	0.82	
CaO	3.27	8.02	7.17	2.92	8.28	2.83	2.78	7.29	8.56	8.95	9.36	9.42	9.60	2.79	2.80	
Na_2_O	5.01	3.07	4.81	4.19	2.94	4.26	4.19	3.74	3.09	3.24	2.98	2.81	2.77	4.33	4.37	
K_2_O	3.05	1.63	1.68	3.24	1.33	3.44	3.27	1.57	1.33	1.31	1.08	1.17	1.17	3.23	3.21	
P_2_O_5_	0.11	0.12	0.19	0.08	0.13	0.08	0.09	0.12	0.12	0.13	0.11	0.13	0.10	0.07	0.08	
Total	100.00	100.00	100.00	100.00	100.00	100.00	100.00	100.00	100.00	100.00	100.00	100.00	100.00	100.00	100.00	
LOI	5.29	1.89	3.44	3.14	1.41	2.35	2.24	1.42	1.42	1.33	1.07	1.33	1.33	2.43	2.38	

Trace Elements (ppm)																
Be	2	1	1	2	1	2	2	1	1	1	1	1	1	2	2	
Sc	5	18	17	4	16	4	4	17	19	17	22	22	24	4	4	
V	39	121	152	36	137	36	34	149	122	145	148	153	158	36	35	
Cr	bdl	40.0	bdl	bdl	bdl	bdl	bdl	bdl	bdl	bdl	30.0	bdl	bdl	bdl	bdl	
Co	5	16	13	4	17	4	4	15	15	19	19	19	19	4	4	
Ni	bdl	bdl	bdl	bdl	bdl	bdl	bdl	bdl	bdl	bdl	bdl	bdl	bdl	bdl	bdl	
Cu	bdl	bdl	bdl	bdl	bdl	10	10	bdl	bdl	10	bdl	bdl	bdl	bdl	bdl	
Zn	70	50	60	40	50	30	40	50	50	50	50	50	50	40	40	
Ga	12	14	15	13	15	13	13	15	14	15	14	14	14	13	13	
Rb	80	37	38	86	24	88	88	40	29	30	24	20	25	87	90	
Sr	303	467	419	280	607	276	277	519	552	639	598	595	504	277	278	
Y	17	16.8	24.6	17.3	15.7	16.4	17.4	18.2	16.2	17.3	15.9	16	16.6	15.7	15.8	
Zr	174	143	197	203	123	200	189	117	139	130	115	116	113	199	190	
Nb	8.9	6.9	10.6	9.9	6.0	9.3	9.4	5.5	7.1	7.0	5.6	6.0	5.2	9.5	9.6	
Cs	2.6	1.0	1.3	2.8	0.6	2.8	2.9	1.1	0.8	0.7	0.5	0.5	0.6	2.8	2.9	
Ba	690	330	332	761	265	780	797	423	294	284	237	234	227	779	798	
La	33	19	22.7	35.9	15.7	34.3	34.4	18.4	16.4	16.8	13.7	13.8	13.4	33.8	34.4	
Ce	56.8	36.3	45.8	57.7	31.3	58	57.9	34.9	33.3	32.8	27.6	27.8	26.7	57.4	57.9	
Pr	5.58	3.96	5.28	5.94	3.65	5.52	5.7	3.93	3.7	3.81	3.21	3.29	3.21	5.46	5.56	
Nd	18.1	15.4	20.7	19.6	14.4	18.1	18.7	15	13.7	15.3	12.9	13.4	12.6	18	18	
Sm	3.15	3.15	4.5	3.34	3.01	3.06	3.11	3.1	2.99	3.29	2.89	3.04	2.76	2.84	2.93	
Eu	0.71	0.86	1.22	0.73	0.92	0.69	0.74	0.95	0.87	0.98	0.90	0.88	0.89	0.65	0.67	
Gd	2.77	3.01	4.44	2.76	2.93	2.56	2.76	3.05	2.87	3.21	2.88	3.14	2.95	2.44	2.57	
Tb	0.44	0.5	0.7	0.45	0.47	0.42	0.45	0.49	0.47	0.53	0.45	0.49	0.49	0.4	0.4	
Dy	2.71	2.95	4.32	2.8	2.79	2.5	2.7	3.04	2.85	3.04	2.69	2.71	2.95	2.49	2.55	
Ho	0.54	0.59	0.85	0.55	0.56	0.55	0.55	0.63	0.55	0.59	0.55	0.56	0.58	0.52	0.52	
Er	1.75	1.73	2.4	1.69	1.56	1.6	1.81	1.86	1.61	1.77	1.56	1.58	1.77	1.61	1.61	
Tm	0.28	0.25	0.38	0.27	0.24	0.26	0.27	0.28	0.24	0.26	0.23	0.24	0.24	0.26	0.25	
Yb	1.93	1.75	2.46	1.91	1.59	1.85	1.95	1.75	1.63	1.71	1.49	1.53	1.6	1.92	1.82	
Lu	0.33	0.29	0.39	0.33	0.25	0.33	0.33	0.30	0.27	0.26	0.24	0.24	0.28	0.34	0.32	
Hf	4.3	3.6	4.9	5.0	3.3	4.7	4.5	3	3.4	3.2	3.1	3.2	3	4.6	4.3	
Ta	1.07	0.58	0.9	1.13	0.51	1.13	1.12	0.49	0.64	0.59	0.48	0.50	0.44	1.14	1.11	
Pb	13	7	8	14	bdl	14	13	8	6	bdl	bdl	6	6	15	14	
Th	10.4	4.64	5.06	11.1	3.19	11	11	3.36	3.94	3.35	2.67	2.73	2.94	10.7	11	
U	3.06	1.29	1.38	3.33	0.89	3.32	3.34	0.95	1.05	0.92	0.71	0.72	0.73	3.24	3.36	
Ratios																
Zr/Ba	0.25	0.43	0.59	0.27	0.46	0.26	0.24	0.28	0.47	0.46	0.49	0.50	0.50	0.26	0.24	
Rb/Sr	0.26	0.08	0.09	0.31	0.04	0.32	0.32	0.08	0.05	0.05	0.04	0.03	0.05	0.31	0.32	
Sr/Ba	0.44	1.42	1.26	0.37	2.29	0.35	0.35	1.23	1.88	2.25	2.52	2.54	2.22	0.36	0.35	
La/SmN	7.33	4.22	3.53	7.52	3.65	7.85	7.74	4.15	3.84	3.57	3.32	3.18	3.40	8.33	8.22	
Tb/YbN	0.93	1.17	1.16	0.96	1.21	0.93	0.94	1.14	1.18	1.27	1.23	1.31	1.25	0.85	0.90	
Eu/Eu*	0.77	0.89	0.88	0.76	1.00	0.78	0.81	1.00	0.96	0.97	1.00	0.92	1.00	0.78	0.77	
Isotope ratios																
^87^Sr/^86^Sr	0.704520	0.704538	nd	nd	0.704256	nd	0.704526	0.704688	0.704551	nd	nd	0.704302	0.704484	nd	nd	
2se	0.000005	0.000005	nd	nd	0.000006	nd	0.000004	0.000006	0.000005	nd	nd	0.000006	0.000007	nd	nd	
^143^Nd/^144^Nd	0.512610	0.512517	nd	nd	0.512545	nd	0.512622	0.512616	0.512506	nd	nd	0.512548	0.512534	nd	nd	
2se	0.000007	0.000008	nd	nd	0.000008	nd	0.000007	0.000007	0.000009	nd	nd	0.000008	0.000008	nd	nd	

Major and trace elements data were performed at the Actalbs Laboratory (Ancaster-Ontario, Canada). Sr Isotope ratios were determined by TIMS Thermo-Finnigan Triton-Ti at the Radiogenic Isotope Laboratory of the Department of Earth Sciences, University of Florence. Nd isotope data were performed at the Radiogenic Isotope Laboratory of the IGG-CNR of Pisa by MC-ICPMS Thermo-Finnigan Neptune-Ti. * Major elements were analysed at the Department of Earth Sciences of the University Florence by XRF and trace elements were analysed at the Department of Earth Sciences of the University of Perugia by ICP-MS (see [8] for analytical details). Italic labels: trace elements analysed by XRF at the Department of Eearth Sciences of the University of Florence (see [6] for analytical details). La/Sm and Tb/Yb ratios are normalised to chondritic values. nd= not determined; bdl= below detection limit; 2se: 2 standard error of the mean.

The pumices are rhyolites (SiO_2_ >70 wt.%) belonging to the high-K calc-alkaline series, whereas the CRC show an affinity with the calc-alkaline series, ranging from basaltic andesite/andesite to dacite (SiO_2_ between 56 and 64 wt.%). Loss on ignition (LOI) is always lower than 2% in the CRCs, while it is up to 5.3% in the pumices.

**Fig. 23:**
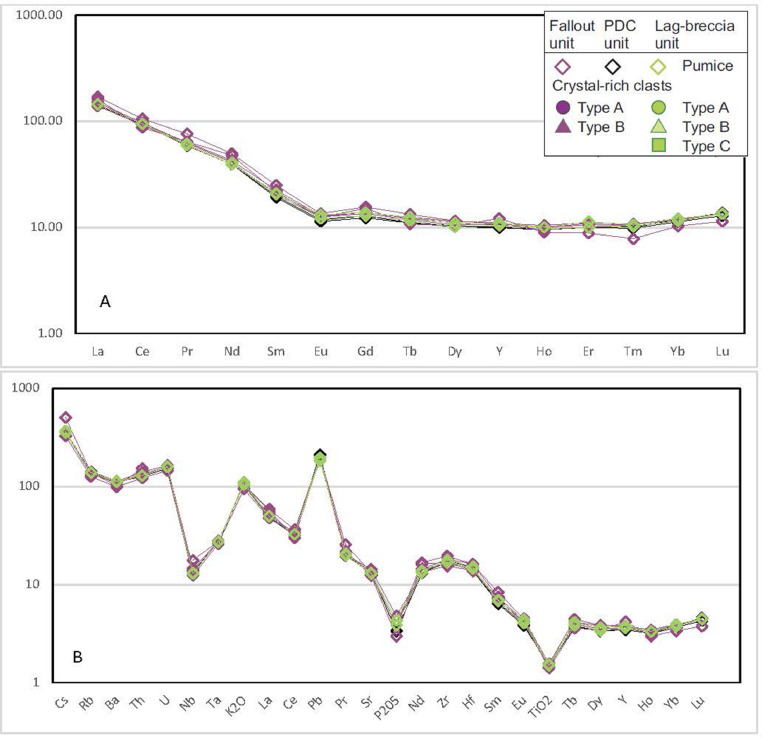
REE (A) and incompatible element (B) patterns of pumice samples.

**Fig. 24:**
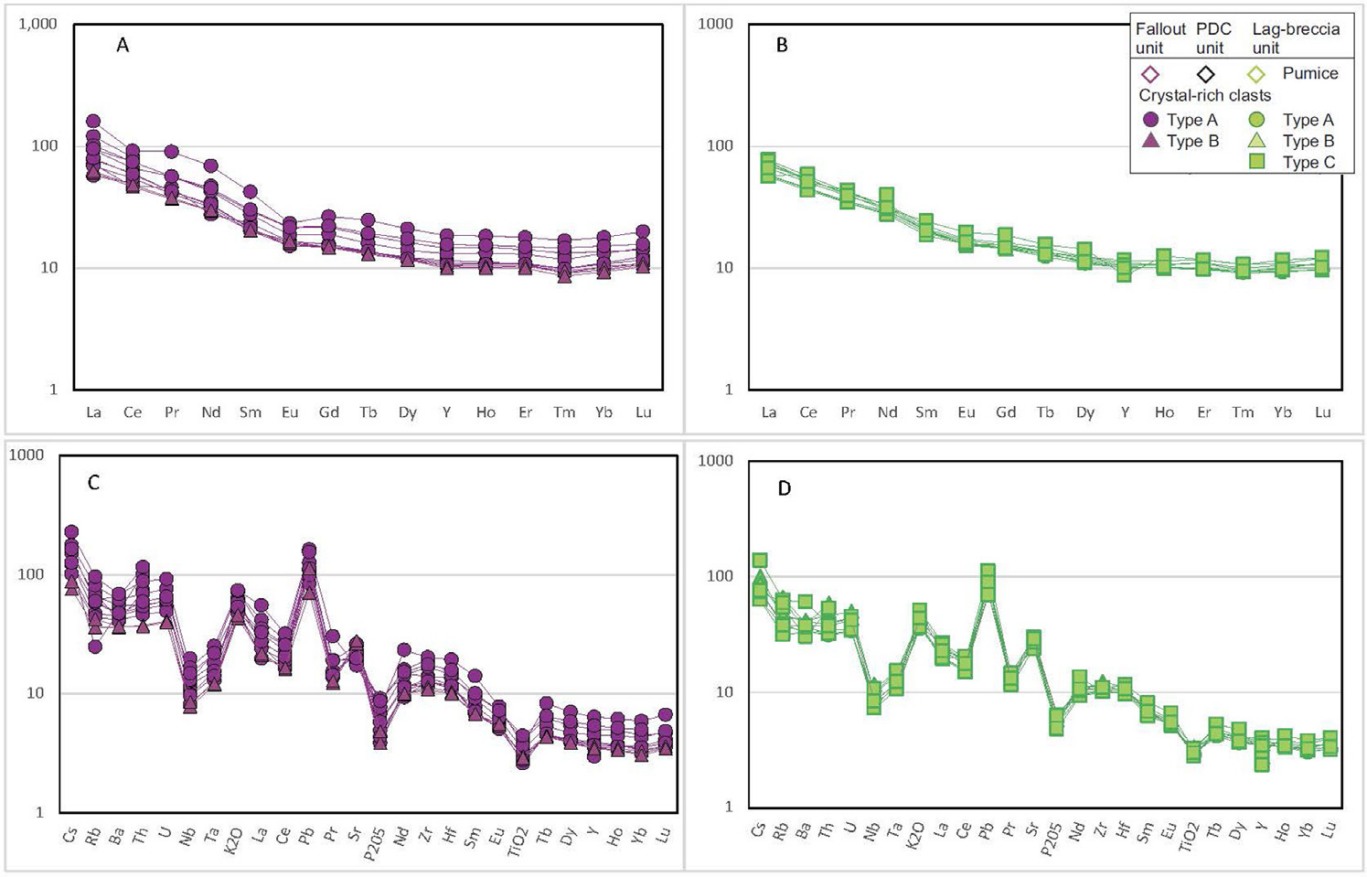
REE (A and B) and incompatible element (C and D) patterns of CRC; A and C are samples from fallout and PDCs deposits, B and D are samples from lag-breccia deposits.

REE and incompatible element patterns (Figs. 23 and 24) are typical for subduction-related calc-alkaline rocks. REE values are normalised to the chondrite data, while incompatible elements are normalised to the primordial mantle values [9]. Symbols used in the graphs are the same used in Braschi et al. [1]: purple symbols represent samples from the fallout deposit, the green ones are samples from the lag-breccia deposit and those from PDC units are black; open diamonds represent pumices, CRCs have different symbols for each texture typology (circles for Type-A, triangles for Type-B and squares for Type-C).

### 1.4 Mineral chemistry

*In situ* investigation of crystal chemistry was also performed on 10 selected samples of pumices and CRCs to explore the minerals and glass compositional variability. The following tables (Table 3, Table 4, Table 5, Table 6, Table 7) report a representative selection of the mineral chemistry composition for plagioclases, pyroxenes, amphiboles and oxides, as well as the composition of glasses.

**Table 3:** Representative major and minor element composition of glasses on selected samples from the Upper Pumice deposit (Nisyros, Greece).

Outcrop	1	1	1	1	1	1	1	1
Depositional unit	Fallout	Fallout	Fallout	Fallout	Fallout	Fallout	Fallout	Fallout
Lithology	Pumice	Pumice	Pumice	Crystal-rich Clast	Crystal-rich Clast	Crystal-rich Clast	Crystal-rich Clast	Crystal-rich Clast
Texture	Porphyritic	Porphyritic	Porphyritic	Type-A	Type-A	Type-A	Type-A	Type-A

Sample	NIS317	NIS317	NIS317	NIS317	NIS317	NIS317	NIS317	NIS317
Oxides wt%								
SiO_2_	74.41	75.42	74.55	75.43	73.83	73.56	74.39	74.15
TiO_2_	0.30	0.33	0.23	0.29	0.21	0.18	0.30	0.25
Al_2_O_3_	11.92	11.99	12.04	12.06	11.78	11.88	11.72	11.63
Cr_2_O_3_	0.02	0.04	bdl	bdl	0.13	bdl	0.02	bdl
FeO	1.27	1.26	1.18	1.67	1.67	1.61	1.67	1.59
MnO	bdl	0.13	0.14	bdl	0.01	0.07	0.08	0.04
MgO	0.16	0.14	0.16	0.18	0.12	0.21	0.20	0.20
CaO	0.91	0.94	0.98	1.02	1.00	1.09	1.07	0.99
Na_2_O	2.82	2.56	2.47	3.12	2.91	3.18	3.02	3.02
K_2_O	4.45	4.40	4.32	4.19	4.15	4.01	4.34	4.18
P_2_O_5_	0.06	bdl	0.06	0.06	bdl	0.05	bdl	0.04
Cl	0.18	0.17	0.28	0.40	0.32	0.28	0.32	0.35
								
Sum	96.50	97.38	96.41	98.42	96.13	96.11	97.14	96.43

Outcrop	1	1	1	1	1	1	1	1
Depositional unit	Fallout	Fallout	Fallout	Fallout	Fallout	Fallout	Fallout	Fallout
Lithology	Crystal-rich Clast	Crystal-rich Clast	Crystal-rich Clast	Crystal-rich Clast	Crystal-rich Clast	Crystal-rich Clast	Crystal-rich Clast	Crystal-rich Clast
Texture	Type-A	Type-A	Type-A	Type-A	Type-B	Type-B	Type-B	Type-B

Sample	NIS357	NIS357	NIS357	NIS357	NIS318	NIS318	NIS318	NIS318
Oxides wt%								
SiO_2_	75.42	74.49	75.52	74.77	74.37	75.04	74.96	74.25
TiO_2_	0.31	0.33	0.50	0.37	0.22	0.18	0.25	0.21
Al_2_O_3_	11.75	11.66	11.31	11.43	11.92	11.98	12.02	12.19
Cr_2_O_3_	0.03	0.05	0.01	0.01	bdl	0.01	bdl	0.05
FeO	1.60	1.28	1.32	1.54	1.33	1.62	1.21	1.29
MnO	bdl	bdl	0.04	0.06	0.06	0.03	0.06	0.03
MgO	0.14	0.21	0.20	0.23	0.14	0.17	0.14	0.17
CaO	1.02	1.13	1.01	0.91	0.92	0.95	0.85	0.88
Na_2_O	3.14	3.35	3.27	2.97	2.78	2.47	2.98	2.92
K_2_O	3.87	3.92	3.82	3.84	4.46	4.04	4.42	4.53
P_2_O_5_	bdl	bdl	0.01	bdl	0.08	bdl	0.04	0.10
Cl	0.27	0.25	0.25	0.26	0.17	0.22	0.19	0.26
								
Sum	97.56	96.66	97.27	96.36	96.45	96.73	97.11	96.91

Outcrop	1	5	5	5	5	5	5	8
Depositional unit	Fallout	Fallout	Fallout	Fallout	Fallout	Fallout	Fallout	Lag-breccia
Lithology	Crystal-rich Clast	Pumice	Pumice	Pumice	Pumice	Pumice	Crystal-rich Clast	Crystal-rich Clast
Texture	Type-B	Porphyritic	Porphyritic	Porphyritic	Porphyritic	Porphyritic	Type-B	Type-A

Sample	NIS318	NIS315	NIS315	NIS315	NIS315	NIS315	NIS316e	NIS420
Oxides wt%								
SiO_2_	73.43	74.54	74.47	76.10	73.30	74.03	74.01	75.74
TiO_2_	0.24	0.19	0.20	0.23	0.21	0.18	0.20	0.30
Al_2_O_3_	11.87	12.19	11.84	12.36	12.46	12.50	12.54	11.67
Cr_2_O_3_	0.05	bdl	bdl	bdl	0.04	bdl	0.05	0.02
FeO	1.45	1.32	1.09	1.21	1.28	1.29	1.24	1.30
MnO	0.04	0.09	bdl	0.03	0.04	0.06	0.02	0.03
MgO	0.13	0.15	0.21	0.17	0.16	0.19	0.18	0.11
CaO	0.87	0.87	0.89	0.98	1.01	0.94	0.88	0.76
Na_2_O	2.96	2.75	2.81	2.71	2.28	2.37	2.33	3.30
K_2_O	4.38	4.24	4.31	4.33	4.46	4.16	4.16	4.26
P_2_O_5_	0.02	bdl	0.07	bdl	0.03	bdl	bdl	0.05
Cl	0.21	0.18	0.21	0.20	0.21	0.17	0.27	0.34
Sum	95.67	96.53	96.09	98.31	95.48	95.89	95.88	97.88
Outcrop	8	8	8	8	8	8	8	8
Depositional unit	Lag-breccia	Lag-breccia	Lag-breccia	Lag-breccia	Lag-breccia	Lag-breccia	Lag-breccia	Lag-breccia
Lithology	Crystal-rich Clast	Crystal-rich Clast	Crystal-rich Clast	Crystal-rich Clast	Crystal-rich Clast	Crystal-rich Clast	Crystal-rich Clast	Crystal-rich Clast
Texture	Type-A	Type-A	Type-A	Type-A	Type-B	Type-B	Type-B	Type-B

Sample	NIS420	NIS420	NIS420	NIS420	NIS424	NIS424	NIS424	NIS424
Oxides wt%								
SiO_2_	75.27	77.41	77.25	76.40	76.84	75.21	76.16	76.42
TiO_2_	0.24	0.29	0.20	0.26	0.20	0.16	0.21	0.21
Al_2_O_3_	11.74	11.92	11.99	11.60	12.37	12.10	12.18	11.91
Cr_2_O_3_	bdl	bdl	0.04	bdl	bdl	0.04	0.05	bdl
FeO	1.18	1.03	0.50	0.94	0.90	0.97	1.25	1.10
MnO	bdl	bdl	bdl	0.03	bdl	bdl	bdl	bdl
MgO	0.20	0.13	0.07	0.11	0.03	0.14	0.13	0.13
CaO	0.83	0.84	0.55	0.83	0.70	0.78	0.71	0.67
Na_2_O	2.81	3.35	2.81	2.97	2.74	3.18	3.25	3.11
K_2_O	4.55	4.37	5.27	4.47	4.75	4.51	4.76	4.79
P_2_O_5_	bdl	0.04	bdl	bdl	bdl	bdl	0.05	bdl
Cl	0.36	0.23	0.09	0.21	bdl	0.29	0.29	bdl
Sum	97.18	99.60	98.76	97.82	98.54	97.37	99.03	98.33

Outcrop	8	8	8	8	8	8	8	8
Depositional unit	Lag-breccia	Lag-breccia	Lag-breccia	Lag-breccia	Lag-breccia	Lag-breccia	Lag-breccia	Lag-breccia
Lithology	Crystal-rich Clast	Crystal-rich Clast	Crystal-rich Clast	Crystal-rich Clast	Crystal-rich Clast	Crystal-rich Clast	Crystal-rich Clast	Crystal-rich Clast
Texture	Type-B	Type-B	Type-B	Type-B	Type-B	Type-C	Type-C	Type-C

Sample	NIS424	NIS430	NIS430	NIS430	NIS430	NIS428	NIS428	NIS428
Oxides wt%								
SiO_2_	74.90	75.70	75.95	75.51	75.11	73.58	76.22	75.72
TiO_2_	0.16	0.24	0.26	0.28	0.31	0.20	0.23	0.24
Al_2_O_3_	12.14	12.17	11.96	11.91	11.93	12.48	12.54	12.75
Cr_2_O_3_	bdl	0.03	bdl	bdl	0.01	bdl	0.08	bdl
FeO	1.08	1.12	0.92	1.19	1.28	1.46	1.47	1.44
MnO	bdl	bdl	bdl	bdl	0.06	bdl	0.05	bdl
MgO	0.13	0.12	0.06	0.13	0.12	0.21	0.19	0.20
CaO	0.73	0.78	0.80	0.72	0.84	1.01	0.82	0.95
Na_2_O	2.71	2.84	3.11	3.08	2.94	3.30	3.10	2.73
K_2_O	4.68	4.35	4.56	4.35	4.49	4.35	4.23	4.19
P_2_O_5_	0.05	0.05	bdl	bdl	bdl	0.07	bdl	0.03
Cl	0.24	0.39	0.37	0.32	0.22	0.25	0.30	0.22
								
Sum	96.81	97.79	97.99	97.48	97.30	96.91	99.22	98.48

Outcrop	8	8						
Depositional unit	Lag-breccia	Lag-breccia						
Lithology	Crystal-rich Clast	Crystal-rich Clast						
Texture	Type-C	Type-C						

Sample	NIS428	NIS428						
Oxides wt%								
SiO_2_	74.47	74.65						
TiO_2_	0.25	0.25						
Al_2_O_3_	12.10	12.48						
Cr_2_O_3_	bdl	bdl						
FeO	1.33	1.04						
MnO	0.04	0.01						
MgO	0.21	0.15						
CaO	0.91	1.06						
Na_2_O	2.83	3.37						
K_2_O	4.50	4.44						
P_2_O_5_	bdl	0.06						
Cl	0.27	0.24						
								
Sum	96.91	97.76						

The composition of glassy groundmasses were obtained with a Jeol JXA 8600 superprobe at the CNR-IGG in Florence. bdl= below detection limit

**Table 4:** Representative plagioclase composition (wt.%) on selected samples from the Upper Pumice deposit (Nisyros, Greece). Plagioclase crystals analysed in the pumice samples are generally more albitic (ca. 30 % An) than those in CRCs (>60 % An).

Outcrop	1	1	1	1	1	1	1	1	1
Depositional unit	Fallout	Fallout	Fallout	Fallout	Fallout	Fallout	Fallout	Fallout	Fallout
Lithology	Banded Pumice	Banded Pumice	Banded Pumice	Banded Pumice	Crystal-rich portion	Crystal-rich portion	Crystal-rich portion	Crystal-rich portion	Crystal-rich clast
Texture	Porphyritic	Porphyritic	Porphyritic	Porphyritic	Type-A	Type-A	Type-A	Type-A	Type-A

Sample	NIS317	NIS317	NIS317	NIS317	NIS317	NIS317	NIS317	NIS317	NIS357

Zone	core	rim	core	rim	core	rim	gdm	gdm	core
SiO_2_	51.81	62.31	60.96	61.31	50.96	59.41	52.34	59.08	50.02
TiO_2_	0.05	0.10	bdl	bdl	0.04	0.01	bdl	bdl	0.03
Al_2_O_3_	30.19	24.16	24.31	24.47	30.80	24.86	29.32	25.09	31.38
Fe_2_O_3_	0.59	0.30	0.33	0.39	0.50	0.40	0.42	0.39	0.56
MgO	0.08	bdl	0.04	bdl	0.08	0.04	0.03	bdl	0.06
CaO	13.89	5.63	6.51	6.44	13.75	7.13	12.36	7.86	14.38
Na_2_O	3.45	7.25	6.82	6.90	3.89	7.10	4.38	6.96	3.57
K_2_O	0.17	0.91	0.75	0.73	0.09	0.53	0.16	0.57	0.13
Sum	100.24	100.66	99.71	100.24	100.10	99.46	99.01	99.95	100.12
FeO	0.530	0.269	0.296	0.354	0.449	0.359	0.380	0.349	0.507
Si	2.352	2.747	2.719	2.719	2.321	2.668	2.398	2.648	2.284
Al	1.616	1.255	1.278	1.279	1.653	1.316	1.583	1.325	1.688
Ti	0.002	0.003	0.000	0.000	0.001	0.000	0.000	0.000	0.001
Fe3+	0.020	0.010	0.011	0.013	0.017	0.013	0.015	0.013	0.019
Mg	0.006	0.000	0.003	0.000	0.005	0.002	0.002	0.000	0.004
Ca	0.676	0.266	0.311	0.306	0.671	0.343	0.607	0.377	0.703
Na	0.304	0.620	0.590	0.593	0.344	0.618	0.389	0.605	0.316
K	0.010	0.051	0.043	0.042	0.005	0.030	0.009	0.032	0.008
Ab	30.71	66.14	62.52	63.08	33.70	62.33	38.71	59.62	30.77
An	68.28	28.39	32.94	32.51	65.81	34.61	60.36	37.18	68.48
Or	1.01	5.48	4.53	4.41	0.49	3.06	0.93	3.20	0.75

Outcrop	1	1	1	1	1	1	1	5	5
Depositional unit	Fallout	Fallout	Fallout	Fallout	Fallout	Fallout	Fallout	Fallout	Fallout
Lithology	Crystal-rich clast	Crystal-rich clast	Crystal-rich clast	Crystal-rich clast	Crystal-rich clast	Crystal-rich clast	Crystal-rich clast	Pumice	Pumice
Texture	Type-C	Type-C	Type-C	Type-A	Type-A	Type-A	Type-A	Porphyritic	Porphyritic

Sample	NIS368c	NIS368c	NIS368c	NIS369a	NIS369a	NIS369a	NIS369a	NIS315	NIS315

Zone	core	rim	core	Core	Core	Core	Rim	core	rim
SiO_2_	49.28	57.14	46.70	48.81	46.26	48.30	59.50	61.52	60.33
TiO_2_	bdl	0.04	0.02	bdl	0.07	0.02	bdl	bdl	bdl
Al_2_O_3_	32.40	28.21	34.66	31.58	32.59	32.85	25.59	24.36	24.30
Fe_2_O_3_	0.60	0.39	0.42	0.60	0.45	0.47	0.53	0.24	0.41
MgO	0.09	0.07	bdl	0.14	0.19	0.10	0.08	0.02	bdl
CaO	15.49	9.55	17.55	15.50	17.47	15.89	7.74	6.02	6.89
Na_2_O	2.29	4.93	1.33	3.20	1.73	2.52	6.12	7.33	6.90
K_2_O	0.15	0.42	bdl	0.11	0.17	bdl	0.56	0.72	0.66
Sum	100.30	100.75	100.67	99.94	98.93	100.14	100.12	100.21	99.49
FeO	0.538	0.355	0.374	0.544	0.406	0.426	0.475	0.220	0.370
Si	2.246	2.539	2.132	2.240	2.157	2.210	2.648	2.725	2.702
Al	1.740	1.478	1.865	1.708	1.791	1.771	1.342	1.272	1.283
Ti	0.000	0.001	0.001	0.000	0.002	0.001	0.000	0.000	0.000
Fe3+	0.020	0.013	0.014	0.021	0.016	0.016	0.018	0.008	0.014
Mg	0.006	0.005	0.000	0.009	0.013	0.007	0.005	0.001	0.000
Ca	0.756	0.454	0.858	0.762	0.873	0.779	0.369	0.286	0.331
Na	0.202	0.425	0.118	0.285	0.156	0.223	0.528	0.630	0.599
K	0.009	0.024	0.000	0.007	0.010	0.000	0.032	0.041	0.038
Ab	20.92	47.03	12.05	26.9	15.0	22.3	56.8	65.80	61.91
An	78.17	50.34	87.95	72.1	84.0	77.7	39.7	29.86	34.16
Or	0.91	2.64	0.00	0.62	0.95	0.00	3.41	4.25	3.90

Outcrop	5	5	5	5	8	8	8	8	8
Depositional unit	Fallout	Fallout	Fallout	Fallout	Lag-breccia	Lag-breccia	Lag-breccia	Lag-breccia	Lag-breccia
Lithology	Pumice	Pumice	Crystal-rich clast	Crystal-rich clast	Crystal-rich clast	Crystal-rich clast	Crystal-rich clast	Crystal-rich clast	Crystal-rich clast
Texture	Porphyritic	Porphyritic	Type-B	Type-B	Type-A	Type-A	Type-A	Type-B	Type-B

Sample	NIS315	NIS315	NIS316e	NIS316e	NIS420	NIS420	NIS420	NIS424	NIS424

Zone	core	rim	core	rim	core	core	gdm	core	core
SiO_2_	61.60	60.66	56.52	57.67	49.79	51.00	52.40	47.32	59.45
TiO_2_	bdl	bdl	bdl	bdl	0.07	0.01	bdl	bsl	0.09
Al_2_O_3_	24.12	24.59	27.74	27.07	30.62	30.53	29.96	33.42	25.12
Fe_2_O_3_	0.12	0.31	0.44	0.27	0.60	0.64	0.50	0.65	0.54
MgO	bdl	bdl	bdl	0.02	0.02	0.14	0.05	0.16	bdl
CaO	5.61	6.23	9.43	8.64	14.09	13.95	13.04	17.27	7.58
Na_2_O	7.61	7.34	6.07	5.96	3.35	3.65	4.79	1.68	6.12
K_2_O	0.82	0.69	0.34	0.44	0.08	0.11	0.12	0.07	0.55
Sum	99.88	99.82	100.54	100.07	98.64	100.02	100.85	100.57	99.44
FeO	0.110	0.280	0.400	0.240	0.544	0.580	0.446	0.583	0.484
Si	2.739	2.703	2.527	2.580	2.303	2.325	2.367	2.165	2.665
Al	1.264	1.291	1.462	1.427	1.670	1.641	1.595	1.802	1.327
Ti	0.000	0.000	0.000	0.000	0.002	0.000	0.000	0.000	0.003
Fe3+	0.004	0.010	0.015	0.009	0.021	0.022	0.017	0.022	0.018
Mg	0.000	0.000	0.000	0.001	0.002	0.009	0.003	0.011	0.000
Ca	0.267	0.297	0.452	0.414	0.698	0.682	0.631	0.847	0.364
Na	0.656	0.634	0.526	0.517	0.301	0.322	0.419	0.149	0.532
K	0.047	0.039	0.019	0.025	0.005	0.006	0.007	0.004	0.031
Ab	67.51	65.13	52.72	54.04	29.95	31.91	39.66	14.94	57.34
An	27.50	30.55	45.26	43.29	69.57	67.48	59.68	84.67	39.27
Or	4.79	4.03	1.94	2.63	0.48	0.61	0.66	0.39	3.39

Outcrop	8	8	8	8	8	8	8	8	8
Depositional unit	Lag-breccia	Lag-breccia	Lag-breccia	Lag-breccia	Lag-breccia	Lag-breccia	Lag-breccia	Lag-breccia	Lag-breccia
Lithology	Crystal-rich clast	Crystal-rich clast	Crystal-rich clast	Crystal-rich clast	Crystal-rich clast	Crystal-rich clast	Crystal-rich clast	Crystal-rich clast	Crystal-rich clast
Texture	Type-B	Type-B	Type-C	Type-C	Type-C	Type-B	Type-B	Type-B	Type-B

Sample	NIS424	NIS424	NIS428	NIS428	NIS428	NIS430	NIS430	NIS430	NIS430

Zone	core	core	core	rim	core	core	core	rim	core
SiO_2_	51.46	49.35	46.92	56.42	48.71	52.03	46.30	46.85	57.63
TiO_2_	0.11	0.06	0.05	bdl	bdl	bdl	bdl	0.10	bdl
Al_2_O_3_	29.70	31.79	32.97	27.06	31.05	30.48	33.67	33.14	25.73
Fe_2_O_3_	0.65	0.49	0.58	0.54	0.88	0.59	0.44	0.61	0.42
MgO	0.07	0.11	0.09	0.03	0.02	0.06	0.01	0.06	0.05
CaO	13.18	15.72	17.23	9.49	15.63	13.30	17.17	17.12	9.17
Na_2_O	4.05	2.75	1.81	5.91	2.73	4.20	1.63	1.82	5.50
K_2_O	0.16	0.05	0.08	0.44	0.12	0.15	0.04	0.03	0.69
Sum	99.37	100.32	99.74	99.90	99.15	100.80	99.26	99.73	99.18
FeO	0.589	0.437	0.523	0.484	0.795	0.529	0.396	0.549	0.382
Si	2.358	2.253	2.166	2.543	2.255	2.350	2.145	2.162	2.606
Al	1.604	1.710	1.794	1.437	1.694	1.623	1.839	1.803	1.371
Ti	0.004	0.002	0.002	0.000	0.000	0.000	0.000	0.003	0.000
Fe3+	0.023	0.017	0.020	0.018	0.031	0.020	0.015	0.021	0.014
Mg	0.005	0.008	0.006	0.002	0.002	0.004	0.001	0.004	0.003
Ca	0.647	0.769	0.853	0.458	0.775	0.643	0.852	0.846	0.444
Na	0.360	0.244	0.162	0.516	0.245	0.368	0.147	0.163	0.482
K	0.009	0.003	0.005	0.026	0.007	0.009	0.002	0.002	0.040
Ab	35.41	23.98	15.91	51.63	23.85	36.05	14.66	16.13	49.89
An	63.70	75.71	83.65	45.81	75.43	63.11	85.10	83.66	45.98
Or	0.89	0.31	0.44	2.55	0.71	0.84	0.24	0.20	4.13

Footnotes: gdm=crystal size <0.5 mm. Ab= Albite, An= Anorthite; Or= Orthoclase.

**Table 5:** Representative pyroxene composition (wt.%) on selected samples from the Upper Pumice deposit (Nisyros, Greece). The pyroxenes are mostly orthopyroxenes; clinopyroxenes are less common and are generally found as microcrystals or in aggregates.

Outcrop	1	1	1	1	1	1	1	1	1	1	1	1	1	1	1	5	5
Depositional unit	Fallout	Fallout	Fallout	Fallout	Fallout	Fallout	Fallout	Fallout	Fallout	Fallout	Fallout	Fallout	Fallout	Fallout	Fallout	Fallout	Fallout
Lithology	Pumice	CRC	CRC	CRC	CRC	CRC	CRC	CRC	CRC	CRC	CRC	CRC	CRC	CRC	CRC	Pumice	Pumice
Texture	Porphyritic	Type-A	Type-A	Type-A	Type-A	Type-A	Type-A	Type-A	Type-A	Type-A	Type-B	Type-B	Type-C	Type-C	Type-C	Porphyritic	Porphyritic

Sample	NIS317	NIS317	NIS317	NIS357	NIS357	NIS368d	NIS368d	NIS369a	NIS369a	NIS369a	NIS318	NIS318	NIS368c	NIS368c	NIS368c	NIS315	NIS315

Phase	opx	opx	opx	opx	opx	opx	opx	opx	opx	cpx	opx	cpx	opx	opx	opx	opx	opx

Zone	core	core	core	core	rim	core	rim	core	core	core	core	core	core	rim	core	core	rim

SiO_2_	52.51	51.02	50.45	55.68	52.20	53.50	51.59	51.42	51.78	51.73	51.12	52.44	53.00	54.25	53.05	52.72	52.99
TiO_2_	0.10	0.23	0.31	0.22	0.25	0.17	0.14	0.09	0.17	0.66	0.21	0.28	0.21	0.09	0.17	0.14	0.13
Al_2_O_3_	0.32	0.75	2.05	1.85	1.83	1.74	0.54	0.29	0.57	1.93	0.93	1.05	0.80	1.80	0.60	0.50	0.32
FeO	25.07	27.20	27.65	11.57	24.32	15.32	25.44	27.63	23.46	12.09	25.95	11.20	19.32	13.11	20.49	23.64	23.81
MnO	1.28	0.93	0.73	0.28	0.66	0.36	0.70	0.79	0.54	0.37	1.19	0.47	0.54	0.34	0.68	1.25	1.16
MgO	20.08	18.36	18.33	28.83	20.28	26.91	20.28	19.00	21.23	13.48	19.41	13.40	24.49	28.84	23.61	20.51	21.02
CaO	1.16	1.21	0.91	1.76	1.16	1.31	0.97	1.26	1.84	19.46	0.92	21.38	1.28	1.74	1.25	1.14	1.02
Na_2_O	0.03	bdl	bdl	bdl	bdl	0.02	0.02	bdl	0.08	0.27	bdl	0.35	0.03	0.11	0.04	0.09	bdl
K_2_O	0.06	0.02	0.02	bdl	0.02	0.02	bdl	0.08	bdl	bdl	bdl	bdl	bdl	0.05	bdl	0.02	bdl
Sum	100.60	99.72	100.45	100.19	100.70	99.37	99.68	100.56	99.67	99.98	99.73	100.56	99.69	100.33	99.88	100.01	100.45

SiO_2_	52.51	51.02	50.45	55.68	52.20	53.50	51.59	51.42	51.78	51.73	51.12	52.44	53.00	54.25	53.05	52.72	52.99
TiO_2_	0.10	0.23	0.31	0.22	0.25	0.17	0.14	0.09	0.17	0.66	0.21	0.28	0.21	0.09	0.17	0.14	0.13
Al_2_O_3_	0.32	0.75	2.05	1.85	1.83	1.74	0.54	0.29	0.57	1.93	0.93	1.05	0.80	1.80	0.60	0.50	0.32
Fe_2_O_3_	1.21	1.42	1.89	bdl	0.02	1.51	2.03	2.89	2.80	0.26	1.78	1.78	1.98	3.12	1.63	0.27	0.31
FeO	23.98	25.93	25.95	11.57	24.30	13.95	23.61	25.04	20.93	11.86	24.35	9.59	17.55	10.30	19.02	23.40	23.53
MnO	1.28	0.93	0.73	0.28	0.66	0.36	0.70	0.79	0.54	0.37	1.19	0.47	0.54	0.34	0.68	1.25	1.16
MgO	20.08	18.36	18.33	28.83	20.28	26.91	20.28	19.00	21.23	13.48	19.41	13.40	24.49	28.84	23.61	20.51	21.02
CaO	1.16	1.21	0.91	1.76	1.16	1.31	0.97	1.26	1.84	19.46	0.92	21.38	1.28	1.74	1.25	1.14	1.02
Na_2_O	0.03	bdl	bdl	bdl	bdl	0.02	0.02	bdl	0.08	0.27	bdl	0.35	0.03	0.11	0.04	0.09	bdl
K_2_O	0.06	0.02	0.02	bdl	0.02	0.02	bdl	0.08	bdl	bdl	bdl	bdl	bdl	0.05	bdl	0.02	bdl
sum	100.72	99.87	100.64	100.22	100.71	99.52	99.88	100.85	99.95	100.02	99.91	100.79	99.89	100.64	100.07	100.06	100.48
Si	1.975	1.957	1.919	1.974	1.953	1.94	1.96	1.952	1.946	1.945	1.948	1.956	1.951	1.923	1.961	1.985	1.985
Al	0.014	0.034	0.081	0.026	0.047	0.06	0.02	0.013	0.025	0.055	0.042	0.044	0.035	0.075	0.026	0.015	0.014
Ti	0.003	0.007	0.000	0.000	0.000	0.00	0.00	0.003	0.005	0.000	0.006	0.000	0.006	0.001	0.005	0.000	0.001
Fe^3+^	0.007	0.003	0.000	0.000	0.000	0.00	0.02	0.033	0.024	0.000	0.005	0.000	0.009	0.000	0.009	0.000	0.000
Al	0.000	0.000	0.011	0.051	0.033	0.01	0.00	0.000	0.000	0.030	0.000	0.002	0.000	0.000	0.000	0.007	0.000
Ti	0.000	0.000	0.009	0.006	0.007	0.00	0.00	0.000	0.000	0.019	0.000	0.008	0.000	0.001	0.000	0.004	0.003
Fe^3+^	0.027	0.038	0.054	0.000	0.001	0.04	0.04	0.050	0.055	0.007	0.046	0.050	0.046	0.083	0.037	0.008	0.009
Mg	1.126	1.050	1.039	1.524	1.131	1.45	1.15	1.075	1.190	0.755	1.102	0.745	1.344	1.524	1.301	1.151	1.174
Fe^2+^	0.755	0.831	0.825	0.343	0.760	0.42	0.75	0.795	0.658	0.373	0.776	0.299	0.540	0.305	0.588	0.737	0.737
Mn	0.041	0.030	0.024	0.008	0.021	0.01	0.02	0.025	0.017	0.012	0.038	0.015	0.017	0.010	0.021	0.040	0.037
Ca	0.047	0.050	0.037	0.067	0.046	0.05	0.04	0.051	0.074	0.784	0.037	0.854	0.051	0.066	0.050	0.046	0.041
Na	0.002	0.000	0.000	0.000	0.000	0.00	0.00	0.000	0.006	0.019	0.000	0.025	0.002	0.008	0.003	0.007	0.000
K	0.003	0.001	0.001	0.000	0.001	0.00	0.00	0.004	0.000	0.000	0.000	0.000	0.000	0.002	0.000	0.001	0.000
En	56.24	52.43	52.50	78.46	57.73	73.43	56.89	53.0	58.9	39.11	54.99	37.96	67.0	76.6	64.9	58.10	58.77
Fe	41.43	45.09	45.61	18.09	39.90	24.01	41.15	44.5	37.4	20.29	43.15	18.54	30.5	20.1	32.6	39.58	39.18
Wo	2.33	2.49	1.88	3.45	2.37	2.56	1.96	2.5	3.7	40.59	1.86	43.50	2.5	3.3	2.5	2.32	2.05
Mg#	0.58	0.54	0.54	0.81	0.59	0.75	0.58	0.54	0.61	0.66	0.56	0.67	0.69	0.79	0.67	0.59	0.60

Outcrop	5	5	8	8	8	8	8	8	8	8	8	8	8				
Depositional unit	Fallout	Fallout	Lag-breccia	Lag-breccia	Lag-breccia	Lag-breccia	Lag-breccia	Lag-breccia	Lag-breccia	Lag-breccia	Lag-breccia	Lag-breccia	Lag-breccia				
Lithology	Pumice	CRC	CRC	CRC	CRC	CRC	CRC	CRC	CRC	CRC	CRC	CRC	CRC				
Texture	Porphyritic	Type-B	Type-A	Type-A	Type-A	Type-A	Type-A	Type-B	Type-B	Type-B	Type-B	Type-C	Type-C				

Sample	NIS315	NIS316e	NIS420	NIS420	NIS420	NIS420	NIS420	NIS424	NIS424	NIS424	NIS430	NIS428	NIS428				

Phase	cpx	opx	cpx	opx	opx	opx	opx	cpx	opx	opx	opx	opx	opx				

Zone	core	core		core	rim	core	rim	core	core	core							

SiO_2_	51.81	50.72	52.87	53.66	52.97	52.63	53.03	52.36	52.27	54.17	52.94	51.73	52.11				
TiO_2_	0.40	0.14	0.30	0.33	0.11	0.24	0.11	0.33	0.15	0.12	0.04	0.21	0.25				
Al_2_O_3_	1.60	0.76	0.93	3.31	0.72	4.67	0.45	1.05	0.75	2.37	0.25	1.58	2.19				
FeO	9.88	27.39	11.34	14.33	23.13	13.10	24.95	10.35	22.43	17.21	23.32	22.62	20.78				
MnO	0.48	1.25	0.29	0.18	0.67	0.23	0.71	0.38	0.85	0.30	0.87	0.65	0.58				
MgO	13.90	18.42	14.31	26.45	21.65	26.87	20.74	13.96	21.54	26.59	21.36	21.59	23.13				
CaO	21.80	0.96	19.98	2.25	0.89	1.70	0.80	21.74	1.37	0.63	1.03	1.23	1.28				
Na_2_O	bdl	bdl	0.24	bdl	bdl	0.04	bdl	0.37	0.13	0.03	bdl	0.07	0.08				
K_2_O	bdl	bdl	bdl	bdl	0.02	bdl	0.01	0.03	bdl	bdl	0.01	bdl	0.04				
Sum	99.87	99.64	100.26	100.52	100.16	99.48	100.82	100.57	99.49	101.42	99.83	99.68	100.45				
SiO_2_	51.81	50.72	52.87	53.66	52.97	52.63	53.03	52.36	52.27	54.17	52.94	51.73	52.11				
TiO_2_	0.40	0.14	0.30	0.33	0.11	0.24	0.11	0.33	0.15	0.12	0.04	0.21	0.25				
Al_2_O_3_	1.60	0.76	0.93	3.31	0.72	4.67	0.45	1.05	0.75	2.37	0.25	1.58	2.19				
Fe_2_O_3_	0.85	2.16	0.38	0.10	0.18	0.47	0.23	2.64	1.53	1.10	0.32	1.78	2.46				
FeO	9.12	25.44	11.00	14.25	22.97	12.68	24.75	7.98	21.06	16.22	23.03	21.01	18.57				
MnO	0.48	1.25	0.29	0.18	0.67	0.23	0.71	0.38	0.85	0.30	0.87	0.65	0.58				
MgO	13.90	18.42	14.31	26.45	21.65	26.87	20.74	13.96	21.54	26.59	21.36	21.59	23.13				
CaO	21.80	0.96	19.98	2.25	0.89	1.70	0.80	21.74	1.37	0.63	1.03	1.23	1.28				
Na_2_O	bdl	bdl	0.24	bdl	bdl	0.04	bdl	0.37	0.13	0.03	bdl	0.07	0.08				
K_2_O	bdl	bdl	bdl	bdl	0.02	bdl	0.01	0.03	bdl	bdl	0.01	bdl	0.04				
sum	99.98	99.90	100.30	100.53	100.17	99.53	100.84	100.83	99.71	101.53	99.88	99.91	100.76				
Si	1.941	1.947	1.974	1.920	1.979	1.890	1.984	1.945	1.961	1.933	1.989	1.936	1.915				
Al	0.059	0.034	0.026	0.080	0.021	0.110	0.016	0.046	0.033	0.067	0.011	0.064	0.085				
Ti	0.000	0.004	0.000	0.000	0.000	0.000	0.000	0.009	0.004	0.000	0.000	0.000	0.000				
Fe^3+^	0.000	0.015	0.000	0.000	0.000	0.000	0.000	0.000	0.002	0.000	0.000	0.000	0.000				
Al	0.012	0.000	0.015	0.060	0.011	0.087	0.004	0.000	0.000	0.033	0.000	0.006	0.009				
Ti	0.011	0.000	0.009	0.009	0.003	0.007	0.003	0.000	0.000	0.003	0.001	0.006	0.007				
Fe^3+^	0.024	0.048	0.011	0.003	0.005	0.013	0.006	0.074	0.042	0.030	0.009	0.050	0.068				
Mg	0.776	1.054	0.796	1.411	1.206	1.438	1.157	0.773	1.205	1.415	1.196	1.205	1.267				
Fe^2+^	0.286	0.817	0.344	0.426	0.718	0.381	0.774	0.248	0.661	0.484	0.723	0.658	0.571				
Mn	0.015	0.041	0.009	0.005	0.021	0.007	0.023	0.012	0.027	0.009	0.028	0.021	0.018				
Ca	0.875	0.039	0.799	0.086	0.035	0.065	0.032	0.865	0.055	0.024	0.042	0.049	0.050				
Na	0.000	0.000	0.017	0.000	0.000	0.003	0.000	0.027	0.009	0.002	0.000	0.005	0.006				
K	0.000	0.000	0.000	0.000	0.001	0.000	0.001	0.001	0.000	0.000	0.001	0.000	0.002				
En	39.28	52.35	40.65	73.05	60.74	75.53	58.06	39.20	60.52	72.12	59.87	60.76	64.18				
Fe	16.44	45.69	18.55	22.49	37.48	21.03	40.33	16.92	36.71	26.66	38.05	36.75	33.27				
Wo	44.28	1.96	40.80	4.47	1.79	3.43	1.61	43.88	2.77	1.22	2.08	2.49	2.55				
Mg#	0.71	0.53	0.69	0.76	0.62	0.78	0.59	0.70	0.62	0.73	0.61	0.62	0.66				

cpx: clinopyroxene; opx: orthopyroxene; bdl = below detection limit; En= Enstatite; Fe= Ferrosilite; Wo= Wollastonite; Mg#: molecular Mg/(Mg+Fe+Mn)

**Table 6:** Representative amphibole composition (wt.%) on selected samples from the Upper Pumice deposit (Nisyros, Greece). Amphiboles are ubiquitous in the CRC samples and rare in pumices.

Outcrop	1	1	1	1	1	8	8	8
Depositional unit	Fallout	Fallout	Fallout	Fallout	Fallout	Lag-breccia	Lag-breccia	Lag-breccia
Lithology	CRC	CRC	CRC	CRC	CRC	CRC	CRC	CRC
Texture	Type-A	Type-A	Type-A	Type-A	Type-A	Type-A	Type-B	Type-C

Sample	NIS357	NIS357	NIS368d	NIS369a	NIS369a	NIS420	NIS424	NIS428

Zone	core	core	core	core	core	core	core	core

SiO_2_	45.23	41.67	42.59	45.09	43.65	42.15	42.49	44.97
TiO_2_	2.04	3.40	2.89	1.88	2.03	2.60	2.51	2.07
Al_2_O_3_	8.22	10.92	9.93	9.12	10.67	11.59	11.33	8.34
FeO	16.18	16.61	14.66	14.41	13.51	14.61	14.39	14.70
MnO	0.24	0.18	0.14	0.23	0.16	0.16	0.28	0.30
MgO	12.26	11.11	13.08	13.69	13.86	12.92	13.09	13.33
CaO	11.02	10.59	10.88	10.67	10.74	10.42	11.14	10.98
Na_2_O	2.00	2.52	2.23	1.73	1.76	2.60	2.14	1.74
K_2_O	0.32	0.40	0.61	0.56	0.47	0.44	0.51	0.41
Cl	0.05	0.07	bdl	0.12	0.17	bdl	0.05	0.12
Sum	97.55	97.47	97.01	97.67	97.01	97.49	97.93	96.97
SiO_2_	45.23	41.67	42.59	45.09	43.65	42.15	42.49	44.97
TiO_2_	2.04	3.40	2.89	1.88	2.03	2.60	2.51	2.07
Al_2_O_3_	8.22	10.92	9.93	9.12	10.67	11.59	11.33	8.34
Fe_2_O_3_	6.60	7.33	8.32	10.26	10.98	10.23	9.18	8.36
FeO	10.24	10.01	7.17	5.18	3.63	5.40	6.13	7.18
MnO	0.24	0.18	0.14	0.23	0.16	0.16	0.28	0.30
MgO	12.26	11.11	13.08	13.69	13.86	12.92	13.09	13.33
CaO	11.02	10.59	10.88	10.67	10.74	10.42	11.14	10.98
Na_2_O	2.00	2.52	2.23	1.73	1.76	2.60	2.14	1.74
K_2_O	0.32	0.40	0.61	0.56	0.47	0.44	0.51	0.41
Cl	0.05	0.07	0.00	0.12	0.17	0.00	0.05	0.12
H_2_O*	2.03	2.01	2.04	2.05	2.03	2.06	2.05	2.02
Sum	100.24	100.21	99.88	100.74	100.14	100.57	100.90	99.88
O=F,Cl	0.01	0.02	bdl	0.03	0.04	bdl	0.01	0.03
Total	100.23	100.20	99.88	100.72	100.10	100.57	100.89	99.86
Si	6.647	6.176	6.268	6.510	6.316	6.131	6.172	6.581
Al iv	1.353	1.824	1.723	1.490	1.684	1.869	1.828	1.419
Al vi	0.071	0.084	0.000	0.062	0.135	0.118	0.110	0.020
Cr	0.000	0.000	0.000	0.018	0.000	0.000	0.000	0.006
Fe3+	0.729	0.818	0.921	1.114	1.196	1.120	1.003	0.921
Fe2+	1.259	1.241	0.882	0.626	0.439	0.657	0.745	0.879
Mn	0.030	0.023	0.017	0.029	0.020	0.019	0.034	0.037
Mg	2.686	2.455	2.869	2.947	2.990	2.802	2.833	2.909
Ca	1.736	1.682	1.715	1.650	1.665	1.624	1.734	1.722
Na	0.569	0.725	0.636	0.484	0.495	0.733	0.604	0.494
K	0.061	0.075	0.114	0.103	0.087	0.081	0.094	0.077
Cl	0.012	0.017	0.000	0.028	0.042	0.000	0.013	0.029
OH*	1.988	1.983	2.000	1.972	1.958	2.000	1.987	1.971

Amphibole names	magnesio-hornblende	ferrian-titanian-tschermakite	ferrian-titanian-tschermakitic hornblende	ferri-magnesio-hornblende	ferri-tschermakitic hornblende	ferri-titanian-tschermakite	ferri-titanian-tschermakite	ferrian-magnesio-hornblende
Texture	Type-C	Type-C	Type-C	Type-B	Type-B	Type-B		

Sample	NIS428	NIS428	NIS428	NIS430	NIS430	NIS430		

Zone	core	core	rim	core	rim	core		

SiO_2_	46.12	42.69	42.98	45.75	41.93	42.50		
TiO_2_	1.43	2.43	2.07	1.69	2.40	2.04		
Al_2_O_3_	8.29	12.75	11.08	8.64	13.03	12.54		
FeO	13.66	9.25	14.82	15.00	11.99	10.88		
MnO	0.27	0.12	0.18	0.38	0.20	0.18		
MgO	14.24	15.57	12.47	13.39	13.88	14.86		
CaO	10.84	11.54	10.94	10.41	10.82	10.75		
Na_2_O	1.54	2.56	2.15	1.74	2.48	1.80		
K_2_O	0.30	0.35	0.44	0.36	0.35	0.35		
Cl	0.04	0.01	0.01	0.15	bdl	bdl		
Sum	96.73	97.28	97.14	97.50	97.07	95.89		
SiO_2_	46.12	42.69	42.98	45.75	41.93	42.50		
TiO_2_	1.43	2.43	2.07	1.69	2.40	2.04		
Al_2_O_3_	8.29	12.75	11.08	8.64	13.03	12.54		
Fe_2_O_3_	9.97	6.85	8.26	10.87	9.59	11.99		
FeO	4.69	3.08	7.38	5.22	3.36	0.08		
MnO	0.27	0.12	0.18	0.38	0.20	0.18		
MgO	14.24	15.57	12.47	13.39	13.88	14.86		
CaO	10.84	11.54	10.94	10.41	10.82	10.75		
Na_2_O	1.54	2.56	2.15	1.74	2.48	1.80		
K_2_O	0.30	0.35	0.44	0.36	0.35	0.35		
Cl	0.04	0.01	0.01	0.15	bdl	bdl		
H_2_O*	2.06	2.09	2.05	2.04	2.08	2.08		
Sum	99.83	100.05	100.04	100.70	100.23	99.28		
O=F,Cl	0.01	bdl	bdl	0.03	bdl	bdl		
Total	99.82	100.05	100.04	100.67	100.23	99.28		
Si	6.671	6.126	6.296	6.602	6.052	6.119		
Al iv	1.329	1.874	1.704	1.398	1.948	1.881		
Al vi	0.083	0.283	0.208	0.071	0.268	0.246		
Cr	0.003	0.000	0.004	0.008	0.014	0.011		
Fe3+	1.085	0.740	0.911	1.180	1.042	1.299		
Fe2+	0.568	0.370	0.904	0.630	0.405	0.010		
Mn	0.034	0.015	0.022	0.047	0.024	0.022		
Mg	3.071	3.330	2.724	2.880	2.987	3.190		
Ca	1.680	1.775	1.717	1.609	1.673	1.658		
Na	0.432	0.713	0.610	0.487	0.693	0.503		
K	0.055	0.064	0.083	0.067	0.065	0.064		
Cl	0.009	0.003	0.002	0.035	0.000	0.000		
OH*	1.991	1.997	1.998	1.965	2.000	2.000		
Amphibole names	ferri-magnesio-hornblende	titanian-magnesio-hastingsite	ferrian-tschermakitic hornblende	ferri-magnesio-hornblende	ferri-titanian-tschermakite	ferri-tschermakite		

bdl = below detection limit; * calculated on stoichiometric basis. Stoichiometric calculation and nomenclature are from [10].

**Table 7:** Oxides composition (wt.%) on selected samples from the Upper Pumice deposit (Nisyros, Greece).

	ILMENITE-HEMATITE				ULVOSPINEL-MAGNETITE		
Outcrop							
Depositional unit	Fallout	Fallout	Fallout	Lag-breccia	Fallout	Lag-breccia	Lag-breccia
Lithology	Crystal-rich Clast	Crystal-rich Clast	Crystal-rich Clast	Crystal-rich Clast	Pumice	Crystal-rich Clast	Crystal-rich Clast
Texture	Type-B	Type-C	Type-A	Type-A	Porphyritic	Type-B	Type-C
Sample	NIS318	NIS368c	NIS368d	NIS420–23	NIS317	NIS424	NIS428
SiO_2_	0.06	0.10	bdl	0.02	0.07	0.06	0.05
TiO_2_	43.55	40.90	47.43	42.60	8.36	7.65	6.45
Al_2_O_3_	0.24	0.25	0.02	0.19	1.56	1.42	1.68
Cr_2_O_3_	bdl	0.01	bdl	0.05	0.03	bdl	0.09
FeO	52.37	53.07	47.58	52.49	84.56	86.12	86.29
MnO	0.70	0.47	0.47	0.46	0.43	0.35	0.38
MgO	2.16	bdl	0.11	2.17	1.10	0.63	0.45
CaO	bdl	0.06	0.13	bdl	0.06	0.01	0.11
Sum	99.08	94.85	95.73	97.97	96.17	96.23	95.50
SiO_2_	0.06	0.10	0.00	0.02	0.07	0.06	0.05
TiO_2_	43.55	40.90	47.43	42.60	8.36	7.65	6.45
Al_2_O_3_	0.24	0.25	0.02	0.19	1.56	1.42	1.68
Cr_2_O_3_	bdl	0.01	bdl	0.05	0.03	bdl	0.09
Fe_2_O_3_	19.67	22.45	10.97	20.56	52.40	53.87	55.31
FeO	34.67	32.87	37.70	33.99	37.41	37.64	36.52
MnO	0.70	0.47	0.47	0.46	0.43	0.35	0.38
NiO	bdl	bdl	0.11	bdl	bdl	bdl	bdl
MgO	2.16	1.96	2.35	2.17	1.10	0.63	0.45
CaO	bdl	0.06	0.13	bdl	0.06	0.01	0.11
sum	101.05	99.05	99.18	100.03	101.42	101.63	101.04
Si	0.002	0.003	0.000	0.001	0.00	0.00	0.00
Al	0.007	0.007	0.000	0.005	0.07	0.06	0.07
Cr	0.000	0.000	0.000	0.001	0.00	0.00	0.00
Fe^3+^	0.367	0.428	0.207	0.388	1.46	1.51	1.56
Ti	0.812	0.780	0.896	0.802	0.23	0.21	0.18
Mg	0.080	0.074	0.088	0.081	0.06	0.03	0.03
Ni	0.000	0.000	0.002	0.000	0.00	0.00	0.00
Fe^2+^	0.719	0.697	0.792	0.712	1.16	1.17	1.14
Mn	0.015	0.010	0.010	0.010	0.01	0.01	0.01
Ca	0.000	0.002	0.004	0.000	0.00	0.00	0.00
TiO_2_	47.37	46.12	50.01	46.98	12.05	11.00	9.50
FeO	41.93	41.21	44.20	41.68	53.95	54.14	53.82
Fe_2_O_3_	10.70	12.66	5.79	11.34	34.00	34.86	36.67
ILM'	81.31	78.22	89.60	80.30	21.76	20.63	17.53
HEM'	18.69	21.78	10.40	19.70	74.53	76.44	79.11

bdl = below detection limit; end-members are calculated following the scheme of [11]: ILM= ilmenite; HEM= hematite; USP=ulvospinel; MT= magnetite

## 2 Experimental Design, Materials and Methods

The field work was carried out with a special care in sampling all of the different juveniles characterising each outcrop of the Upper Pumice deposits. A total of 67 samples (Table 1) was collected during two field campaign in 2006 and 2014.

Pumices were sampled from each location with the aim to investigate the possible variability within the evolved juvenile component for a total of 16 samples (Fig. 1). The CRCs were also sampled in detail, collecting 51 samples, according to their evident textural and physical variability (i.e., density, colour, crystal content) to explore their recurrence and distribution among the different outcrops.

During preparation all specimens were cut in order to remove altered portions, then grinded and powdered in an agate mill.

Major and trace elements were analysed by Actalabs Laboratories (Ontario, Canada) using the Lithogeochemistry-4Lithoresearch analytical package. The procedure consists in a lithium metaborate/tetraborate fusion digestion and analyses are carried out using ICP-OES for major elements and ICP-MS for trace elements (see www.actlabs.com). Accuracy and precision for major elements are estimated as better than 3% for Si, Ti, Fe, Ca, and K, and 7% for Mg, Al, Mn, Na; for trace elements (above 10 ppm) they are better than 10%. REE, Rb, Sr, Y, Zr, Hf, Nb, Th, and U were analysed.

Selected powdered samples were processed in an ultraclean laboratory environment (class 1000) at the Department of Earth Sciences of the University Florence. They were preliminarily treated with 2 mL diluted 1 N HCl in an ultrasonic bath for 15′, twice, then rinsed three times with Milli-Q water to minimise isotopic variation induced by supergene processes that could overprint the magmatic signature (e.g. [12] and references therein). After that they were processed using the standard digestion technique described in [13] consisting in a sequential addition of concentrated HF and HNO_3_ (in proportion of 4:1) of suprapure quality, followed by a double addition of concentrated HNO_3_, and subsequently by some 10 mL of diluted 6 N HCl and placed on a hot plate at 140°. Cation-exchange AGW and Ln-spec reusable resins were used for Sr, REE and Nd purification respectively, by sequential addition of properly diluted HCl suprapure acid, as described in Avanzinelli et al. [13].

Sr isotope ratios were determined at the Department of Earth Sciences of the University Florence using the Thermal Ionization Thermo-Finnigan Triton-TI mass spectrometer (TIMS), equipped with nine collectors coupled with nine exchangeable amplifiers. For measurements with the thermal ionization mass spectrometer, 100–150 ng of sample were loaded on single Re-filament as nitrate form, with TaCl_5_ and H_3_PO_4_ as activator and to keep the signal stable during the analyses. ^87^Sr/^86^Sr were measured dynamically using the amplifier rotation method and corrected using an exponential mass fractionation law to ^87^Sr/^86^Sr=0.1194. Each ratio is the average of 120 measurements, to reach good precision (2se) of the data. Within run, replicate measurements of international NIST SRM 987 standard (0.710251 ± 0.000011 [13]) gave mean values of ^87^Sr/^86^Sr = 0.710252 ± 0.000011 (2sd, *n* = 5) well comparable with those reported in literature [14]. All errors reported are 2se (2 standard error of the mean) for single data precisions and 2sd (2 standard deviation) for standards reproducibility (Table 8). The Sr analytical blank, measured during the course of the analytical session, is 60 pg, which is in agreement with blank reproducibility of the lab.

**Table 8:** Accuracy and reproducibility of Sr-Nd isotopes measurements on international and internal reference standards.

Method	Multi-dynamic collection mode		
Isotope	Sr ISOTOPE		
Within run standard			
		**87Sr/86Sr**	**2se**
	**SRM987**	0.710258	± 0.000005
	**SRM987**	0.710245	± 0.000006
	**SRM987**	0.710252	± 0.000005
	**SRM987**	0.710255	± 0.000006
	**SRM987**	0.710248	± 0.000005
			**2sd**
	**Average**	0.710252	0.000011

Reference	***Thirlwall, 1991***	87Sr/86Sr	2sd
		**0.710248**	**0.000011**

Instrument	MULTICOLLECTOR INDUCTIVELY COUPLED PLASMA MASS SPECTROMETER - NEPTUNE PLUS		

Method	**Static collection mode**		
Isotope	**Nd ISOTOPE**		

Within run standard			
		**143Nd/144Nd**	**2se**
	**NdFi**	0.511464	± 0.000006
	**NdFi**	0.511451	± 0.000007
	**NdFi**	0.511466	± 0.000006
	**NdFi**	0.511460	± 0.000007
			**2sd**
	**Average**	0.511460	0.000014

Reference	***Avanzinelli et al., 2005***	143Nd/144Nd	2sd
		**0.511467**	**0.000008**

2se= 2 standard error of the mean; 2sd= 2 standard deviation

Nd isotope ratios were measured in the Laboratory of Radiogenic Isotopes at the IGG-CNR of Pisa using the new Thermo-Finnigan multicollector inductively coupled plasma mass spectrometer (MC-ICP-MS) Neptune-Plus, equipped with a combined cyclonic and Scott-type quartz spray chamber, Ni-cones, a MicroFlow PFA 100 µl/min self-aspiring nebuliser and a Teledyne Cetac ASX-560 Autosampler. All samples were diluted in ultrapure 2% HNO3 solution after digestion and elemental separation. During Nd analyses, instrumental mass fractionation was corrected using the ^146^Nd/^144^Nd ratio (0.7219). Mass interference correction was performed using the ratios ^147^Sm/^144^Sm (4.838710), and ^147^Sm/^148^Sm (1.327400). The analytical accuracy and reproducibility for the within run internal standard NdFi is 0.511460 ±14 (2sd, *n* = 4), well comparable to the average value reported in [13] measured by TIMS. Long-term external reproducibility of the laboratory for ^143^Nd/^144^Nd on international reference material J-Ndi-1 was 0.512098 ± 5 (average of 17 replicates), which match well the reference values of [15], (Table 8).

A number of 10 samples were selected, on the basis of their textural and compositional representativeness, among the different juvenile types (pumices and CRCs) for mineral chemistry investigations on minerals and glasses. Analyses were performed by electron microprobe JEOL Superprobe JXA-8600 at the IGG-CNR of Florence. Working conditions were 15 kV of accelerating voltage and 10 µA of beam current. Beam diameter varied from 2 to 5 µm for mineral phases and 10 for glasses. Peak counting time was 15 sec for major elements, except for Na that is counted for 10 sec to minimize the alkali loss effect, and 40 sec for minor elements. Backgrounds were counted at specific positions for 5 and 20 sec on major and minor elements, respectively.

A set of natural phases (Astimex Albite, Olivine, Diopside, Orthoclase, Plagioclase, Sanidine, Kaersutite, Bustamite, Obsidian and Smithsonian Anorthite *Great Sitikin Islands*, Olivine *San Carlos*, Augite *Kakanui*, Pyrope *Kakanui*, Horneblende *Kakanui*, Ilmenite or Bio-Rad Albite and Orthoclase) and synthetic internal glass standards (ALV-47 and CFA-981) was used as primary and quality control standards. PAP software was used for correction [16]. Precision was within 1% for silica, 2–3% for other major elements and about 5–8% for minor elements.

Scanning Electron Microprobe (SEM) images were achieved at the MEMA laboratory of the University of Florence using 20 kV of acceleration voltage and 2 nA of probe current.

## CRediT authorship contribution statement

**F. Mastroianni:** Investigation, Writing – review & editing, Visualization. **E. Braschi:** Conceptualization, Investigation, Resources, Data curation, Writing – original draft, Visualization. **M. Casalini:** Methodology, Validation, Writing – review & editing. **S. Agostini:** Methodology, Validation. **S. Di Salvo:** Methodology, Validation. **G. Vougioukalakis:** Investigation, Resources, Writing – review & editing. **L. Francalanci:** Writing – review & editing, Supervision, Project administration, Funding acquisition.

## Declaration of Competing Interest

The authors declare that they have no known competing financial interests or personal relationships which have, or could be perceived to have, influenced the work reported in this article.

## Data Availability

Data on the juvenile products of the Upper Pumice eruption on Nisyros (Braschi et al., 2022) (Original data) (Earth/Chem).
